# A cardiovascular disease policy model: part 2—preparing for economic evaluation and to assess health inequalities

**DOI:** 10.1136/openhrt-2014-000140

**Published:** 2016-06-10

**Authors:** K D Lawson, J D Lewsey, I Ford, K Fox, L D Ritchie, H Tunstall-Pedoe, G C M Watt, M Woodward, S Kent, M Neilson, A H Briggs

**Affiliations:** 1 Health Economics and Health Technology Assessment, Institute of Health & Wellbeing, University of Glasgow, Glasgow, UK; 2 Centre for Health Research, School of Medicine, Western Sydney University, Sydney, New South Wales, Australia; 3 Robertson Centre for Biostatistics, Institute of Health & Wellbeing, University of Glasgow, Glasgow, UK; 4 BHF Centre for Research Excellence, University of Edinburgh, Edinburgh, UK; 5 Centre of Academic Primary Care, University of Aberdeen, Aberdeen, UK; 6 Institute of Cardiovascular Research, Ninewells Hospital, University of Dundee, Dundee, UK; 7 General Practice & Primary Care, Institute of Health & Wellbeing, University of Glasgow, Glasgow, UK; 8 The George Institute for Global Health, University of Sydney, Sydney, New South Wales, Australia; 9 Oxford Martin School, University of Oxford, Oxford, UK

**Keywords:** CORONARY ARTERY DISEASE, QUALITY OF CARE AND OUTCOMES

## Abstract

**Objectives:**

This is the second of the two papers introducing a cardiovascular disease (CVD) policy model. The first paper described the structure and statistical underpinning of the state-transition model, demonstrating how life expectancy estimates are generated for individuals defined by ASSIGN risk factors. This second paper describes how the model is prepared to undertake economic evaluation.

**Design:**

To generate quality-adjusted life expectancy (QALE), the Scottish Health Survey was used to estimate background morbidity (health utilities) and the impact of CVD events (utility decrements). The SF-6D algorithm generated utilities and decrements were modelled using ordinary least squares (OLS). To generate lifetime hospital costs, the Scottish Heart Health Extended Cohort (SHHEC) was linked to the Scottish morbidity and death records (SMR) to cost each continuous inpatient stay (CIS). OLS and restricted cubic splines estimated annual costs before and after each of the first four events. A Kaplan-Meier sample average (KMSA) estimator was then used to weight expected health-related quality of life and costs by the probability of survival.

**Results:**

The policy model predicts the change in QALE and lifetime hospital costs as a result of an intervention(s) modifying risk factors. Cost-effectiveness analysis and a full uncertainty analysis can be undertaken, including probabilistic sensitivity analysis. Notably, the impacts according to socioeconomic deprivation status can be made.

**Conclusions:**

The policy model can conduct cost-effectiveness analysis and decision analysis to inform approaches to primary prevention, including individually targeted and population interventions, and to assess impacts on health inequalities.

**Key questions**

**What is already known about this subject?**

- Cardiovascular disease (CVD; rheumatic heart diseases, hypertensive diseases, ischaemic heart diseases, pulmonary heart disease, other forms of heart disease, cerebrovascular diseases, diseases of arteries and diseases of veins) is a leading cause of premature mortality, morbidity and health service costs. The incidence of CVD has also been shown to be socially patterned. The primary prevention of CVD is a policy priority, including the objective to reduce health inequalities. A policy model is a model capable of evaluating effectiveness and cost-effectiveness of a wide range of interventions aimed at modifying known CVD risk factors.

**What does this study add?**

- This policy model joins together risk estimation, individual patient decision-making and societal policymaking in a cohesive whole. By taking a competing risk approach, the model estimates the impact of risk factor modification on CVD events and non-CVD mortality to predict (quality-adjusted) life expectancy, and lifetime health service costs. By including a measure of socioeconomic deprivation as an independent risk factor, the policy model can be used to assess the impact of interventions on health inequalities.

**How might this impact on clinical practice?**

- This policy model uses the ASSIGN risk factors to estimate CVD risk and can project the impact of risk factor modification on (quality-adjusted) life expectancy and lifetime health service costs. Consequently, the model can be used consistently as a clinical and policy tool, to help prioritise individuals for intervention and to evaluate the effectiveness and cost-effectiveness of interventions.

## Introduction

This paper is part 2 of a twinned set of papers introducing an alternative cardiovascular disease (CVD) policy model. The first paper described the structure of the state-transition model and its statistical underpinning, where individuals enter free of CVD and using the ASSIGN risk factor variables, which includes a measure of socioeconomic deprivation, the model estimates life expectancy.^1^ This second paper builds on that approach to detail how the model is prepared to undertake economic evaluation to assess the cost-effectiveness of interventions and the impact on health inequalities.

‘All models are wrong, but some are useful’ (George Box). In developing a model, it is important that the outputs generated are consistent with the needs of decision-makers, and that the methodological approach follows best practice guidance. Reimbursement agencies, such as the National Institute for Health and Care Excellence (NICE) and the Scottish Medicines Consortium (SMC), provide guidance on undertaking cost-effectiveness analysis of new interventions. Key recommendations include that quality-adjusted life years (QALYs) can be generated to account for potential morbidity impacts, the impact on health service costs is estimated net of intervention costs, and that a full uncertainty analysis is undertaken.^2^

The International Society for Pharmacoeconomics and Outcomes Research (ISPOR) recently produced guidance for developing, validating and disseminating models to undertake cost-effectiveness analysis for use in decision analysis.^3^ This is intended to be generalisable for the evaluation of health technology and public health interventions.

There are existing policy models capable of undertaking cost-effectiveness analysis, and several have been used in practice.^4^ ^5^ Models have been built using specific national population data and are not necessarily generalisable internationally. A systematic review of policy models found that most focus on coronary heart disease (CHD) only, are built using cross-sectional data, can lack transparency in the modelling process, conduct limited uncertainty analysis and there is a lack of validation tests regarding outputs produced.^4^

Key reasons for developing this policy model include building a model following recent guidelines and to use the risk factors in the ASSIGN CVD risk equation, thereby aligning the clinical tool currently used in Scotland to screen and prioritise individuals for intervention^6^ ^7^ with a new policy model that can be used to evaluate the cost-effectiveness of interventions to modify risk. Further, by using ASSIGN, which includes a measure of socioeconomic deprivation, a potential novelty would be to evaluate impacts of interventions on reducing health inequalities, which is the principal rationale of primary prevention in Scotland.^6^ Consequently, the overarching intention of building this policy model was to attempt to integrate CVD risk estimation, individual patient decision-making and societal policymaking into a cohesive whole.

The specific objectives of this second paper are to build on the first paper by detailing how life expectancy estimates are quality adjusted (morbidity adjusted) to generate quality-adjusted life expectancy (QALE) and how individuals accumulate health service costs over estimated lifetimes. The term QALE is used hereon, rather than QALY, for exposition purposes to be consistent with the first paper. The paper then illustrates how the model can estimate the cost-effectiveness of individual and population interventions aimed at modifying risk factors by assessing the change in QALE and lifetime health service costs, and estimate the impact on health inequalities.

## Methods

**Figure 1:**
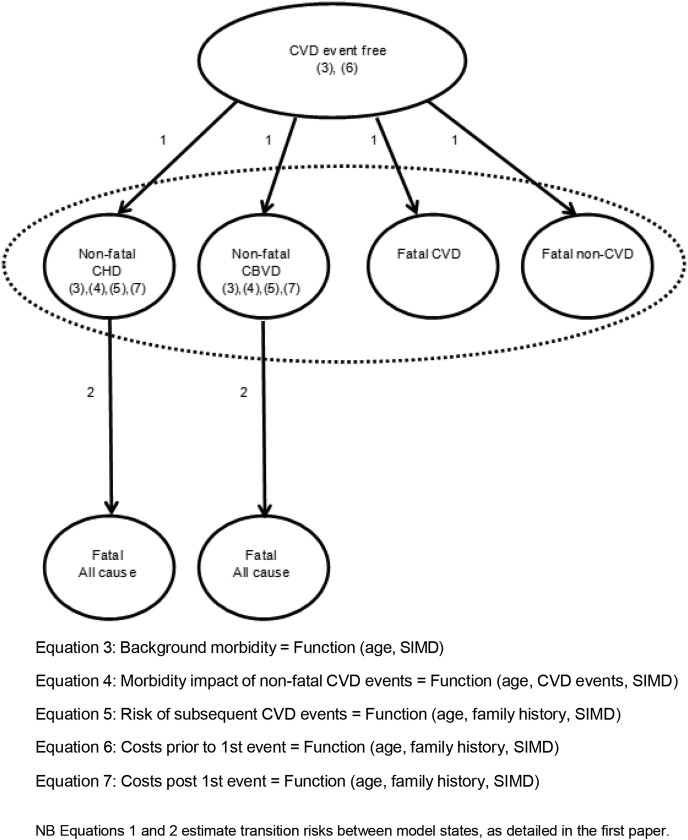
Structure of CVD policy model—quality adjusting life expectancy and estimating lifetime health service costs. CBVD, cerebrovascular disease; CHD, coronary heart disease; CVD, cardiovascular disease; SIMD, Scottish Index of Multiple Deprivation.

A description of methods is given below with technical details available in the online supplementary appendix. Figure 1 illustrates the structure of the state-transition policy model which cycles annually. The first paper described the model in detail and demonstrated how CVD-free individuals, defined by the ASSIGN risk factor variables, enter and transit within the model resulting in estimates of life expectancy specific to individual risk profiles. Separate model equations were estimated for men and women while retaining the same model structure. This paper builds on these survival equations to quality-adjust life expectancy and attach costs to individuals across expected lifetimes.

10.1136/openhrt-2014-000140.supp1 Supplementary appendix

### The process of quality adjusting life expectancy

While an individual may be alive, they are unlikely to be in perfect health. There are three elements of quality adjustment: background morbidity, the impact of experiencing a first non-fatal CVD event and the impact of experiencing subsequent non-fatal CVD events prior to death.

*Background morbidity—health-related quality of life (HRQoL) scores* (see online supplementary equation 3): The Scottish Health Survey (SHeS) 2003,^8^ a representative cross-sectional survey of the general population, included the SF-12 as a measure of HRQoL.^9^ A total of 7054 respondents aged 20 years plus fully completed the SF-12, a response rate of 91% with a slight under-representation of the most socioeconomically deprived fifth in the population (defined below). No bias was expected in the modelled results. Applying the SF-6D algorithm to individuals' SF-12 responses generated a single score representing overall (preference-weighted) HRQoL.^10^ Scores can range from 0.29 (worst health) to 1 (perfect health) on a linear scale. These HRQoL scores represent background morbidity in the general population, also known as ‘population norms’ or ‘health utilities’. Specific estimates were modelled by sex, seven age group categories and fifths of socioeconomic deprivation. Regarding age, seven categories were chosen to be consistent with previous estimates made for the UK:^11^ 20–25, 25–34, 35–44, 45–54, 55–64, 65–74 and 75 plus. Regarding socioeconomic deprivation, the Scottish Index of Multiple Deprivation (SIMD) was used, a measure developed by the Scottish Government. SIMD is an aggregated measure of material deprivation for each household in Scotland and is derived from 37 indicators in seven domains (income, employment, health, education, access to services, housing and crime) and is determined at datazone level (geographical areas with a population of 769).^12^ Each household is given a score and the general population is divided into fifths (quintiles) consisting of an equal number of households. Estimates are provided within the online supplementary appendix.

As individuals enter the policy model, a HRQoL score is automatically selected according to age, sex and SIMD. The model cycles annually and the score updates when an individual enters a new age category. The score is used as a weighting factor to ‘quality-adjust’ an individual's survival probability. This process occurs within the CVD event-free state, the two non-fatal CVD event states and as an individual transits towards a death state.

*Impact of experiencing a first non-fatal CVD event—reducing HRQoL scores* (see online supplementary equation 4): The SHeS 2003 was also used to estimate the impact of experiencing (self-reported) non-fatal CVD events on reducing HRQoL scores. These estimates are known as ‘utility decrements’. The SHeS included four CVD events: myocardial infarction, stroke, irregular heartbeat and intermittent claudication. Using ordinary least squares, the impact of all four events was estimated (see online supplementary appendix). The SHeS did not include heart failure; however, this was considered an important event, and the associated utility decrement estimated by Clarke^13^ was used as the only available estimate that we were aware of. If the first event experienced was non-fatal CHD or non-fatal cerebrovascular disease (CBVD), the model selects the utility decrements of myocardial infarction and stroke, respectively, to further quality-adjust survival.

*Impact of experiencing subsequent non-fatal CVD events—further reducing HRQoL scores* (see online supplementary equations 4 and 5): From a first non-fatal CVD event, an individual is at risk of subsequent non-fatal CVD events prior to death. The Scottish Heart Health Extended Cohort (SHHEC) was linked to Scottish morbidity and death records (SMR) to generate a dataset of over 16 000 individuals followed for an average of 21 years (detailed in the first paper). Probit regression was used to estimate the annual probability of incurring any of five non-fatal CVD events independently, following a first non-fatal CHD or CBVD event, and by using restricted cubic spline functions, event risks were extrapolated beyond the observed follow-up period. Modelled events include CHD, stroke, irregular heartbeat, intermittent claudication and heart failure (see online supplementary appendix).

The probability of a specific event occurring is multiplied by its associated utility decrement, and summed to generate an overall ‘composite utility decrement’. This is used to further quality-adjust an individual's survival. This process is a modified version of the Kaplan-Meier Sample Average (KMSA) estimator approach^14^ ^15^ given event risks are modelled. For exposition purposes, we use the term KMSA estimator hereon. Finally, to generate QALE, the area under the quality-adjusted survival curve is calculated using the trapezoid method with half-cycle correction.

**Figure 2:**
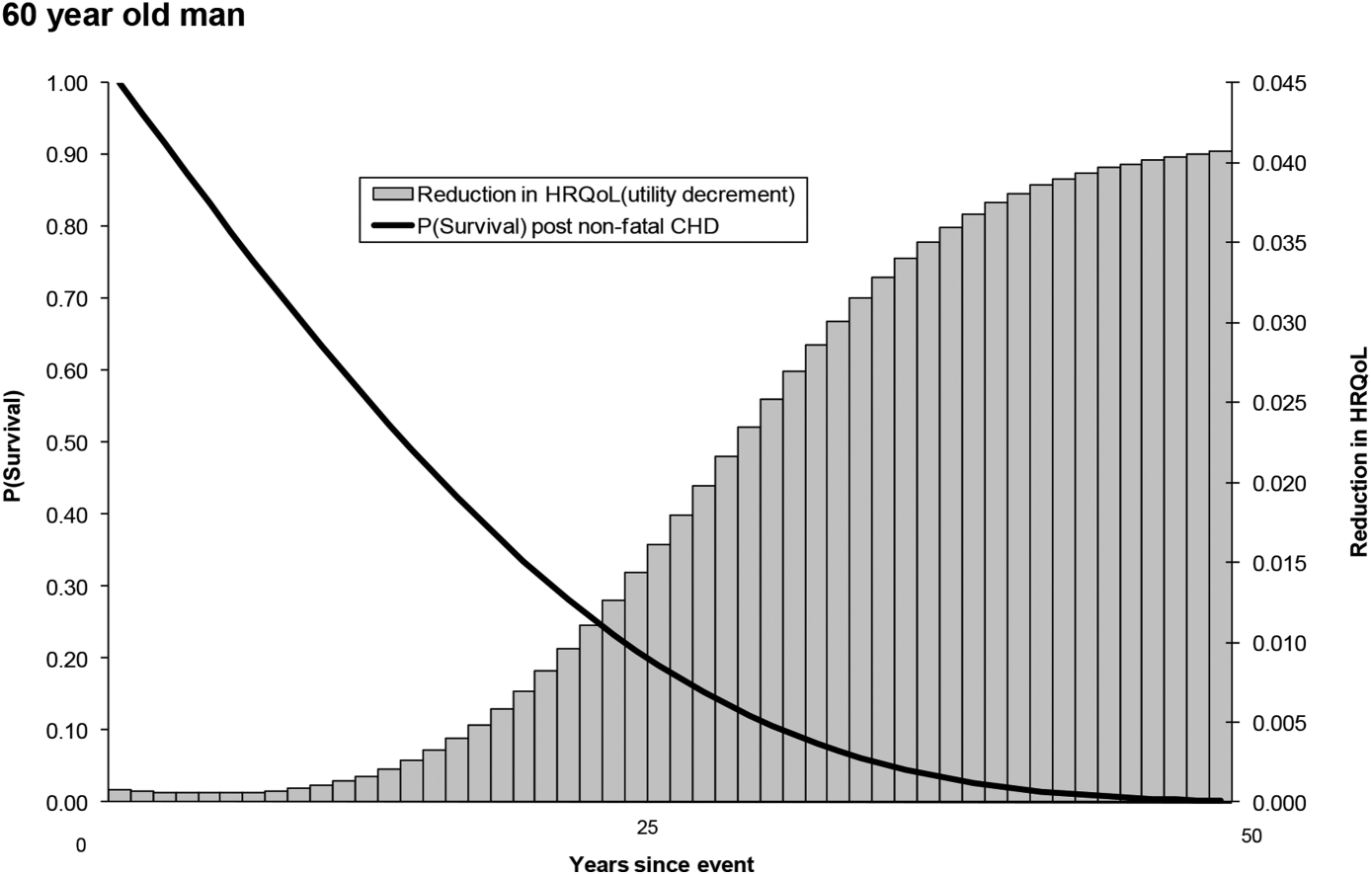
Process of quality adjustment. CHD, coronary heart disease; HRQoL, health-related quality of life.

This quality adjustment process is illustrated for an individual risk profile, defined as follows: a man aged 60 years, post-CHD event, no family history of CVD, non-diabetic, SIMD score of 60.8 (highest fifth of socioeconomic deprivation), systolic blood pressure of 160 mm Hg, total cholesterol of 7 mmol/L, high-density lipoprotein (HDL) cholesterol of 1 mmol/L and smokes 20 cigarettes per day (figure 2).

### Generating QALE

On entering the model, an individual is at risk of the first four events (non-fatal CHD, non-fatal CBVD, fatal CVD and fatal non-CVD), one of which must occur within 100 annual cycles. Crucially, the type and timing of the first event define a different scenario resulting in specific estimates of survival, life expectancy and so QALE. In effect, there are 400 possible scenarios (4 first events×100 model cycles). To generate overall, expected, QALE, the probability of each scenario occurring (taken from the cumulative incidence of the first four events; see the first paper) is multiplied by its associated QALE estimate, and summed.

The policy model discriminates between individual risk profiles to produce specific estimates of event risks, survival, life expectancy and QALE. For illustration, we generated specific QALE for multiple individuals defined by standard 10-year risk tables.^16^ Separate tables are estimated for men and women, for the lowest and highest fifths of SIMD, and by smoking status with the assumption of 20 cigarettes per day for smokers. All risk profiles were attributed an average family history (proportion=0.26) and diabetes (proportion=0.15) derived from SHHEC. This was for exposition purposes to avoid producing additional tables for individuals with and without family history, and with and without diabetes, given journal restrictions on the number of tables. In principle, the model can take into account all ASSIGN risk factors when estimating QALE and health service costs, producing specific estimates, charts and tables as required.

### Estimating lifetime health service costs

The linked SHHEC-SMR dataset recorded all hospitalisations, both CVD and non-CVD related. These were costed using method 1 in Geue,^17^ and the total cost of a continuous inpatient stay (CIS) was estimated. This is driven by the principal cause of admission and additional ‘hotelling’ costs if the observed length of stay exceeded the ‘trim point’ of the principal event. Costs are accumulated in all annual cycles of the model (see online supplementary equations 6 and 7), before the first event and following a non-fatal event.

These estimates are applied in the model by using the same KMSA estimator approach used to generate QALE. In this case, annual costs are weighted by the survival probability of an individual, and the area under the curve is summed to estimate cumulative lifetime costs. This process is illustrated using the same 60-year male profile as before.

### Generating expected lifetime health service costs

To reiterate, an individual faces 400 scenarios on entering the model defined by the type and timing of the first event (4 first events×100 model cycles). Each scenario results in a different lifetime cost estimate. To generate overall, expected, lifetime costs, the probability of each scenario occurring (taken from the cumulative incidence of the first four events) is multiplied by its associated cost estimate, and summed. For consistency, we illustrate the model’s ability to discriminate by estimating lifetime costs for multiple individuals defined by 10-year risk tables.

### Preparing model to be used for cost-effectiveness analysis

The model can be used to estimate the cost-effectiveness of interventions aimed at modifying the ASSIGN risk factors. Potential interventions may include pharmaceuticals, lifestyle interventions and legislative changes. Three main inputs are used in the model: (1) the cost of the intervention(s) which may include one-off costs (eg, legislation) or periodic costs (eg, pharmaceuticals); (2) the associated evidence (efficacy or effectiveness) regarding the interventions' impact on reducing risk factors and (3) relevant adherence/compliance assumptions where necessary, which can be tailored to particular individual risk profiles, such as age, sex and socioeconomic deprivation.

First, preintervention individual risk profiles are run through the model to estimate baseline life expectancy, QALE and costs. Second, risk profiles are adjusted postintervention (using trial evidence or conducting a ‘what-if’ analysis) and individuals are rerun through the model. The difference in life expectancy, QALE and costs (net of intervention costs) is then calculated to generate an incremental cost-effectiveness ratio. Discount rates can be applied according to guidance. For instance, the model currently discounts life expectancy, QALE and costs at a rate of 3.5% for health technology interventions and 1.5% for public health interventions. This approach follows guidance from NICE.^2^ ^18^ ^19^

The model can also incorporate evidence relating to the impact of intervention(s) on event rates (eg, CHD). To do this, the model estimates the necessary changes to relevant risk factors to obtain reported event rates. This can be done using appropriate literature and expert opinion. Further, by converting event rates into assumed risk factor reductions, the model is then able to estimate the impact of interventions on all first four events (non-fatal CHD, non-fatal CBVD, fatal CVD and fatal non-CVD).

*Uncertainty analysis:* There is an uncertainty regarding predictions of event risks, life expectancy, quality adjustment and costs. The model is capable of undertaking a full uncertainty analysis,^3^ ^20^ ^21^ including probabilistic sensitivity analysis (PSA) by varying all parameters at once; an analysis of extremes by taking the limits of the CIs to produce expected, best-case and worst-case scenarios; or a simple one-way sensitivity analysis by varying one parameter at a time. Further, the model can directly vary the discount rate as part of a scenario analysis. The online supplementary appendix details parameter estimates that enable uncertainty analysis to be undertaken, including the variance–covariance relationship between the ASSIGN risk factors and events, and the associated Cholesky decomposition matrixes used in PSA.

*Example case study:* To illustrate the readiness of the model to undertake cost-effectiveness analysis, the paper builds on an example from the first paper. The SHeS 2009 was used to estimate average risk profiles for men and women aged 60 years across fifths of socioeconomic deprivation. The first paper generated undiscounted life expectancy predictions. This exercise was repeated by running the same profiles through the model to estimate QALE and costs. We then conducted a ‘what-if’ exercise, where individuals switch to ‘perfect’ risk factor profiles defined in reference to 10-year risk tables as non-smokers, systolic blood pressure of 100 and a ratio of total cholesterol to HDL cholesterol of 3. For illustrative purposes, it is assumed that individuals immediately switch risk profiles and acquire the associated life expectancy and QALE. In practice, the model can accommodate evidence regarding reversibility of risk and lag effects for instance. The illustration then estimates the maximum theoretical benefit on life expectancy, QALE and lifetime hospital costs if individuals switched to a perfect risk profile. Mean estimates are provided, both undiscounted and discounted.

## Results

### Generating QALE and lifetime costs

Figure 2 illustrates the KMSA estimator approach when quality adjusting survival for a man aged 60 years following a non-fatal CHD event, as defined under the Methods section. The composite utility decrement (ie, the amount by which HRQoL falls due to expected further non-fatal CVD events) is shown in shaded bars. This increases over time, reflecting the increasing probability of incurring CVD events as an individual ages. Annual estimates are used to weight an individual's survival probability, which declines over time.

**Figure 3:**
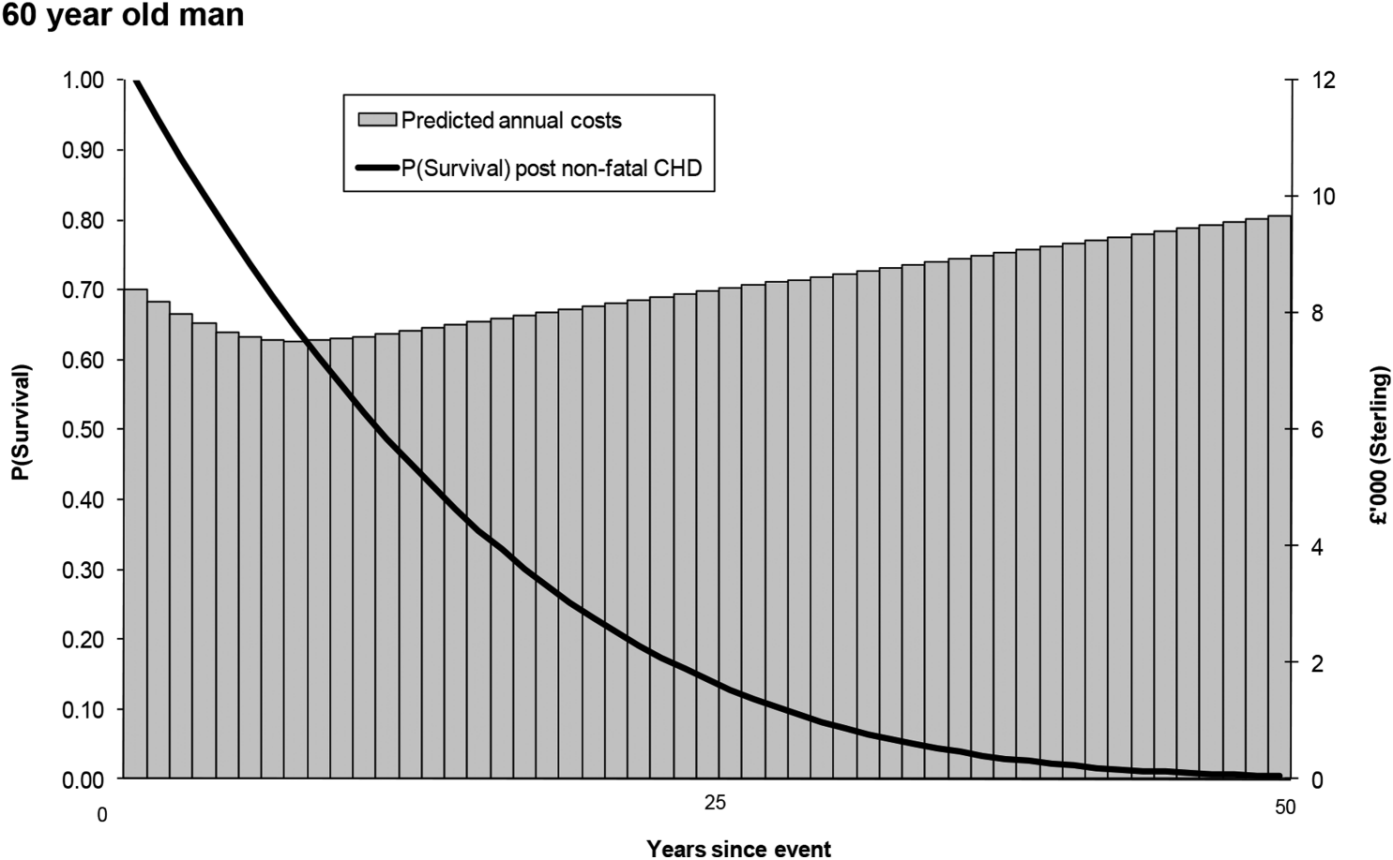
Process of attaching costs. CHD, coronary heart disease.

Figure 3 illustrates a similar approach to generate lifetime costs for the same man aged 60 years. Costs initially decrease postevent as the surviving individual requires less care, and then steadily increase as comorbidities accumulate with age. Annual estimates are weighted by an individual's survival curve.

Table 1 illustrates discounted QALE using the risk profiles of 10-year risk tables and adjusts for all three elements of quality of life: background morbidity, the impact of experiencing a first non-fatal CVD event and the impact of experiencing subsequent non-fatal CVD events. The table is divided into four segments (a–d) representing sex and socioeconomic deprivation status (highest and lowest fifths), with further division by smoking status and four 10-year age bands from 40 to 70 years. The ratios of total cholesterol to HDL cholesterol are represented along the ‘x-axis’ and systolic blood pressure values are represented along the ‘y-axis’.

**Table 1:** Discounted quality-adjusted life expectancy (QALE) for men and women for least and most socioeconomically deprived

		Men—least deprived fifth												Women—least deprived fifth									
		Non-smoker					Smoker							Non-smoker					Smoker				
		*Age 70 years*					*Age 70 years*							*Age 70 years*					*Age 70 years*				
Systolic blood pressure (mm Hg)	180	80.9	80.7	80.7	80.6	80.6	78.8	78.8	78.9	78.9	78.9	Systolic blood pressure (mm Hg)	180	81.9	81.8	81.6	81.6	81.5	79.1	79.1	79.0	79.1	79.1
	160	81.4	81.3	81.3	81.2	81.1	79.2	79.3	79.4	79.4	79.4		160	82.5	82.5	82.3	82.2	82.2	79.7	79.7	79.7	79.7	79.7
	140	81.8	81.7	81.8	81.7	81.6	79.6	79.7	79.8	79.9	79.9		140	83.1	83.0	82.9	82.8	82.7	80.3	80.4	80.3	80.3	80.3
	120	82.1	82.2	82.2	82.1	82.1	79.9	80.1	80.2	80.3	80.3		120	83.7	83.6	83.4	83.3	83.3	80.9	80.9	80.9	80.9	80.9
	100	82.4	82.5	82.6	82.5	82.5	80.1	80.4	80.6	80.6	80.7		100	84.1	84.1	83.9	83.8	83.8	81.3	81.5	81.4	81.4	81.4
		*Age 60 years*					*Age 60 years*							*Age 60 years*					*Age 60 years*				
	180	74.8	74.6	74.6	74.5	74.5	72.8	72.8	72.9	72.9	72.9		180	75.6	75.5	75.3	75.3	75.3	72.9	72.9	72.9	72.9	73.0
	160	75.2	75.1	75.1	75.0	74.9	73.2	73.3	73.3	73.4	73.4		160	76.1	76.1	75.9	75.8	75.8	73.5	73.6	73.5	73.5	73.6
	140	75.6	75.5	75.5	75.4	75.4	73.5	73.6	73.8	73.8	73.8		140	76.6	76.6	76.4	76.3	76.3	74.1	74.2	74.1	74.1	74.1
	120	75.9	75.9	75.9	75.8	75.8	73.8	74.0	74.1	74.2	74.2		120	77.1	77.0	76.8	76.8	76.7	74.6	74.7	74.6	74.6	74.6
	100	76.1	76.2	76.2	76.2	76.1	74.0	74.3	74.5	74.5	74.5		100	77.4	77.4	77.3	77.2	77.2	75.0	75.1	75.1	75.1	75.1
		*Age 50 years*					*Age 50 years*							*Age 50 years*					*Age 50 years*				
	180	68.2	68.1	68.1	68.0	68.0	66.5	66.6	66.7	66.8	66.8		180	68.8	68.7	68.6	68.6	68.6	66.5	66.6	66.6	66.6	66.7
	160	68.6	68.5	68.5	68.4	68.4	66.9	67.0	67.1	67.1	67.2		160	69.2	69.1	69.0	69.0	69.0	67.1	67.1	67.1	67.1	67.2
	140	68.9	68.9	68.8	68.8	68.7	67.2	67.3	67.5	67.5	67.5		140	69.6	69.5	69.4	69.4	69.4	67.6	67.6	67.6	67.6	67.6
	120	69.2	69.2	69.2	69.1	69.1	67.5	67.6	67.8	67.8	67.8		120	70.0	69.9	69.8	69.7	69.7	67.9	68.0	68.0	68.0	68.0
	100	69.4	69.4	69.4	69.3	69.3	67.6	67.9	68.0	68.1	68.0		100	70.3	70.2	70.1	70.0	70.0	68.3	68.4	68.4	68.4	68.4
		*Age 40 years*					Age 40 years							*Age 40 years*					*Age 40 years*				
	180	61.1	61.0	61.0	60.9	60.9	59.8	59.9	59.9	60.0	60.0		180	61.4	61.3	61.3	61.2	61.2	59.7	59.7	59.8	59.8	59.8
	160	61.4	61.3	61.2	61.2	61.2	60.1	60.1	60.3	60.3	60.3		160	61.7	61.6	61.6	61.5	61.5	60.1	60.1	60.1	60.2	60.2
	140	61.6	61.5	61.5	61.5	61.4	60.3	60.4	60.5	60.6	60.6		140	62.0	61.9	61.9	61.8	61.8	60.4	60.5	60.5	60.5	60.5
	120	61.8	61.7	61.7	61.7	61.7	60.5	60.6	60.7	60.8	60.8		120	62.2	62.2	62.1	62.0	62.0	60.7	60.8	60.8	60.8	60.8
	100	61.9	61.9	61.9	61.9	61.9	60.6	60.8	60.9	60.9	60.9		100	62.5	62.4	62.3	62.3	62.2	61.0	61.1	61.0	61.0	61.1
		3	5	7	9	10	3	5	7	9	10			3	5	7	9	10	3	5	7	9	10
					Total/HDL cholesterol ratio												Total/HDL cholesterol ratio						

		Men—most deprived fifth												Women—most deprived fifth									
		Non-smoker					Smoker							Non-smoker					Non-Smoker				
		*Age 70 years*					*Age 70 years*							*Age 70 years*					*Age 70 years*				
Systolic blood pressure (mm Hg)	180	78.8	78.6	78.5	78.4	78.3	76.8	76.8	76.8	76.7	76.7	Systolic blood pressure (mm Hg)	180	80.4	80.3	80.2	80.1	80.1	77.9	77.9	77.9	77.9	77.9
	160	79.3	79.1	79.0	78.9	78.8	77.2	77.2	77.2	77.2	77.1		160	81.0	80.9	80.7	80.6	80.6	78.4	78.5	78.4	78.4	78.4
	140	79.6	79.5	79.5	79.4	79.3	77.5	77.6	77.6	77.6	77.6		140	81.5	81.4	81.3	81.1	81.1	78.9	79.0	78.9	78.9	78.9
	120	79.9	79.9	79.9	79.9	79.8	77.7	77.9	78.0	78.0	78.0		120	82.0	81.9	81.7	81.6	81.6	79.4	79.5	79.4	79.4	79.4
	100	80.1	80.3	80.3	80.2	80.2	77.9	78.1	78.3	78.3	78.3		100	82.5	82.4	82.2	82.1	82.0	79.8	79.9	79.8	79.8	79.8
		*Age 60 years*					*Age 60 years*							*Age 60 years*					*Age 60 years*				
	180	72.6	72.3	72.2	72.1	72.0	70.5	70.4	70.4	70.4	70.4		180	74.2	74.1	73.9	73.8	73.8	71.7	71.7	71.6	71.7	71.7
	160	73.1	72.8	72.7	72.6	72.5	70.9	70.9	70.9	70.9	70.9		160	74.7	74.6	74.4	74.3	74.3	72.2	72.2	72.2	72.2	72.2
	140	73.4	73.3	73.2	73.1	73.0	71.2	71.3	71.4	71.3	71.3		140	75.2	75.1	74.9	74.8	74.7	72.7	72.7	72.7	72.7	72.7
	120	73.8	73.7	73.7	73.5	73.4	71.5	71.7	71.7	71.7	71.7		120	75.6	75.5	75.3	75.2	75.2	73.1	73.2	73.1	73.1	73.1
	100	74.0	74.0	74.0	73.9	73.9	71.7	71.9	72.1	72.1	72.1		100	76.0	75.9	75.7	75.6	75.5	73.5	73.6	73.5	73.5	73.5
		*Age 50 years*					*Age 50 years*							*Age 50 years*					*Age 50 years*				
	180	66.3	66.0	65.9	65.8	65.7	64.4	64.3	64.4	64.3	64.3		180	67.6	67.5	67.4	67.3	67.3	65.4	65.4	65.4	65.4	65.5
	160	66.7	66.4	66.4	66.2	66.1	64.8	64.8	64.8	64.8	64.7		160	68.0	67.9	67.8	67.7	67.6	65.9	65.9	65.9	65.9	65.9
	140	67.0	66.8	66.8	66.6	66.6	65.1	65.1	65.2	65.2	65.1		140	68.4	68.3	68.1	68.1	68.0	66.3	66.3	66.3	66.3	66.3
	120	67.3	67.2	67.1	67.0	67.0	65.4	65.5	65.5	65.5	65.5		120	68.7	68.6	68.4	68.4	68.3	66.6	66.7	66.7	66.7	66.7
	100	67.5	67.5	67.5	67.4	67.3	65.5	65.7	65.8	65.8	65.8		100	69.0	68.9	68.8	68.7	68.6	67.0	67.1	67.0	67.0	67.0
		*Age 40 years*					*Age 40 years*							*Age 40 years*					*Age 40 years*				
	180	59.4	59.2	59.1	59.0	59.0	58.0	57.9	57.9	57.9	57.9		180	60.4	60.3	60.2	60.2	60.2	58.7	58.8	58.7	58.8	58.8
	160	59.8	59.6	59.5	59.4	59.3	58.3	58.2	58.3	58.2	58.2		160	60.7	60.6	60.5	60.5	60.4	59.1	59.1	59.1	59.1	59.1
	140	60.0	59.9	59.8	59.7	59.6	58.5	58.5	58.6	58.6	58.5		140	61.0	60.9	60.8	60.7	60.7	59.4	59.5	59.4	59.4	59.4
	120	60.3	60.2	60.1	60.0	59.9	58.7	58.8	58.8	58.8	58.8		120	61.3	61.2	61.1	61.0	60.9	59.7	59.7	59.7	59.7	59.7
	100	60.4	60.4	60.4	60.2	60.2	58.9	59.0	59.1	59.1	59.0		100	61.5	61.4	61.3	61.2	61.2	59.9	60.0	60.0	60.0	60.0
		3	5	7	9	10	3	5	7	9	10			3	5	7	9	10	3	5	7	9	10
					Total/HDL cholesterol ratio												Total/HDL cholesterol ratio						

HDL, high-density lipoprotein.

The tables illustrate several gradients where, for an otherwise identical risk factor profile, discounted QALE are higher for women compared with men, non-smokers compared with smokers, least deprived fifth compared with the most deprived fifth and for older compared with younger individuals (due to conditional life expectancy).

Table 2 follows a similar format and illustrates that higher discounted costs are associated with healthier risk profiles given longer life expectancies and the accumulation of comorbidities. Several gradients are evident with costs higher for women, the least deprived and younger individuals (due to higher cumulative life expectancy). Smokers in the age groups of 40 and 50 years tend to have higher costs with the expectation of early onset of related health-related conditions.

**Table 2:** Discounted lifetime hospital costs for men and women for least and most socioeconomically deprived

		Men—least deprived fifth												Women—least deprived fifth									
		Non-smoker					Smoker							Non-smoker					Smoker				
		*Age 70 years*					*Age 70 years*							*Age 70 years*					*Age 70 years*				
Systolic blood pressure (mm Hg)	180	46.4	44.8	44.5	44.6	44.7	41.6	41.5	42.1	42.7	43.1	Systolic blood pressure (mm Hg)	180	50.4	49.0	47.9	48.1	48.1	41.7	41.6	41.5	41.9	42.2
	160	48.1	45.9	45.2	45.0	45.0	43.1	42.7	43.0	43.4	43.7		160	52.9	50.6	49.0	48.9	48.7	44.4	43.7	43.3	43.4	43.7
	140	49.7	47.1	46.0	45.4	45.3	44.5	43.8	43.8	44.0	44.2		140	55.5	52.4	50.2	49.7	49.4	47.2	45.9	44.8	44.8	45.0
	120	51.5	48.5	47.0	46.0	45.8	45.9	44.9	44.8	44.6	44.7		120	58.5	54.5	51.7	50.5	50.2	50.3	48.3	46.6	46.3	46.1
	100	53.2	49.9	48.2	46.9	46.5	47.2	46.2	45.7	45.4	45.2		100	61.9	57.0	53.4	51.7	51.3	53.3	50.8	48.6	47.7	47.6
		*Age 60 years*					*Age 60 years*							*Age 60 years*					*Age 60 years*				
	180	53.5	52.4	52.6	53.3	53.7	52.2	52.6	53.7	54.9	55.6		180	56.4	55.9	55.4	56.0	56.6	51.7	52.0	52.2	53.1	53.8
	160	54.5	52.5	52.2	52.5	52.8	53.2	52.9	53.6	54.5	55.0		160	58.1	56.5	55.4	55.6	56.1	53.8	53.4	53.1	53.7	54.3
	140	55.7	52.9	52.0	51.9	52.0	54.3	53.4	53.5	54.1	54.4		140	60.1	57.3	55.5	55.3	55.4	56.2	54.8	53.9	54.1	54.6
	120	57.2	53.6	52.1	51.6	51.5	55.6	54.0	53.6	53.8	54.0		120	62.6	58.5	55.8	55.3	55.1	58.7	56.4	54.9	54.7	55.0
	100	58.8	54.7	52.7	51.6	51.4	56.9	54.8	54.0	53.8	53.8		100	65.5	60.0	56.7	55.4	55.2	61.5	58.2	56.0	55.5	55.4
		*Age 50 years*					*Age 50 years*							*Age 50 years*					*Age 50 years*				
	180	55.1	55.0	56.0	57.5	58.4	57.7	59.0	60.7	62.6	63.7		180	57.0	57.4	57.8	59.2	60.1	56.5	57.4	58.3	59.8	60.8
	160	55.4	54.2	54.5	55.6	56.3	58.0	58.2	59.4	61.0	61.9		160	57.7	56.9	56.8	57.7	58.6	57.7	57.9	58.0	59.2	60.2
	140	56.0	53.7	53.4	53.9	54.4	58.5	57.7	58.3	59.4	60.1		140	59.0	56.8	55.9	56.4	57.1	59.3	58.2	57.9	58.7	59.5
	120	57.2	53.7	52.7	52.7	52.9	59.4	57.7	57.5	58.1	58.6		120	60.7	57.2	55.5	55.5	55.7	61.3	59.0	57.9	58.3	58.8
	100	58.6	54.2	52.5	51.9	51.9	60.6	57.9	57.2	57.2	57.5		100	62.8	58.1	55.4	54.8	54.9	63.4	60.2	58.3	58.1	58.5
		*Age 40 years*					*Age 40 years*							*Age 40 years*					*Age 40 years*				
	180	52.0	53.2	54.9	57.1	58.3	57.5	59.8	62.2	64.8	66.2		180	53.0	54.7	56.0	58.0	59.3	55.7	57.8	59.5	61.6	62.7
	160	51.6	51.5	52.6	54.4	55.4	57.1	58.1	59.9	62.1	63.3		160	53.0	53.5	54.1	55.9	57.0	56.1	57.2	58.2	60.1	61.2
	140	51.8	50.4	50.8	52.0	52.8	57.0	56.9	57.9	59.6	60.7		140	53.5	52.7	52.6	53.8	54.8	57.0	56.8	57.2	58.7	59.5
	120	52.4	49.8	49.4	50.1	50.7	57.5	56.1	56.4	57.6	58.4		120	54.5	52.3	51.5	52.2	52.8	58.1	56.8	56.3	57.3	58.1
	100	53.6	49.8	48.6	48.7	49.0	58.4	55.9	55.4	56.0	56.5		100	56.1	52.4	50.9	51.0	51.3	60.0	57.2	56.0	56.4	57.0
		3.0	5.0	7.0	9.0	10.0	3.0	5.0	7.0	9.0	10.0			3	5	7	9	10	3	5	7	9	10
					Total/HDL cholesterol ratio												Total/HDL cholesterol ratio						

		Men—most deprived fifth												Women—most deprived fifth									
		Non-smoker					Smoker							Non-smoker					Smoker				
		*Age 70 years*					*Age 70 years*							*Age 70 years*					*Age 70 years*				
Systolic blood pressure (mm Hg)	180	40.3	37.8	36.8	36.2	35.9	34.4	33.5	33.3	33.2	33.3	Systolic blood pressure (mm Hg)	180	49.7	47.7	46.2	45.8	45.9	41.4	40.9	40.6	40.8	41.0
	160	42.3	39.4	38.1	37.2	36.8	36.1	34.9	34.6	34.3	34.3		160	52.3	49.4	47.4	46.6	46.5	43.9	42.8	41.9	42.0	42.1
	140	44.3	41.1	39.5	38.3	37.8	37.7	36.3	35.9	35.4	35.3		140	55.4	51.6	48.8	47.5	47.3	46.7	44.9	43.5	43.2	43.0
	120	46.2	42.9	41.0	39.6	39.0	39.2	37.9	37.1	36.6	36.2		120	58.9	53.8	50.4	48.8	48.2	49.7	47.3	45.3	44.4	44.4
	100	48.1	44.8	42.7	41.0	40.3	40.6	39.3	38.4	37.7	37.4		100	62.5	56.8	52.6	50.4	49.7	52.7	49.7	47.3	46.2	45.9
		*Age 60 years*					*Age 60 years*							*Age 60 years*					*Age 60 years*				
	180	49.2	46.7	45.9	45.7	45.7	46.2	45.2	45.3	45.6	45.9		180	58.4	56.6	55.7	55.9	56.3	53.8	53.7	53.4	54.3	54.6
	160	50.9	47.6	46.3	45.7	45.6	47.7	46.1	45.8	45.8	46.0		160	60.3	57.4	55.7	55.5	55.6	55.8	54.6	54.0	54.5	54.7
	140	52.7	48.7	46.9	45.9	45.7	49.2	47.2	46.5	46.2	46.2		140	62.5	58.6	56.1	55.3	55.1	58.1	56.1	54.7	54.6	54.8
	120	54.7	50.2	47.9	46.5	46.0	50.8	48.5	47.4	46.7	46.6		120	65.5	60.3	56.9	55.6	55.1	61.0	57.9	55.8	55.2	55.2
	100	56.7	51.8	49.2	47.4	46.8	52.4	49.8	48.4	47.5	47.1		100	69.0	62.4	58.2	56.2	55.6	64.2	60.2	57.4	56.1	56.0
		*Age 50 years*					*Age 50 years*							*Age 50 years*					*Age 50 years*				
	180	53.3	51.5	51.4	51.9	52.3	54.1	53.7	54.4	55.4	55.9		180	60.8	60.4	60.4	61.5	62.4	60.9	61.6	62.3	63.8	64.6
	160	54.3	51.4	50.7	50.8	51.0	55.1	53.7	53.8	54.4	54.9		160	61.8	60.0	59.2	59.8	60.5	62.1	61.7	61.7	62.7	63.4
	140	55.6	51.8	50.4	50.0	50.0	56.3	54.0	53.5	53.7	54.0		140	63.5	60.1	58.5	58.5	58.7	63.8	62.2	61.2	61.9	62.4
	120	57.4	52.6	50.5	49.6	49.4	57.7	54.7	53.6	53.3	53.3		120	65.8	60.9	58.3	57.7	57.6	66.2	63.0	61.3	61.2	61.7
	100	59.4	53.8	51.1	49.7	49.2	59.3	55.6	54.0	53.3	53.1		100	68.5	62.4	58.7	57.4	57.2	68.8	64.6	61.9	61.2	61.4
		*Age 40 years*					*Age 40 years*							*Age 40 years*					*Age 40 years*				
	180	52.3	51.8	52.6	53.9	54.8	56.7	57.3	58.7	60.5	61.5		180	58.1	59.2	60.2	62.3	63.6	61.9	63.8	65.4	67.8	69.1
	160	52.6	50.8	50.9	51.8	52.5	56.8	56.3	57.0	58.4	59.3		160	58.3	57.9	58.1	59.7	60.6	62.3	62.9	63.8	65.7	66.7
	140	53.3	50.4	49.7	50.1	50.6	57.5	55.7	55.8	56.7	57.3		140	59.1	57.2	56.5	57.4	58.2	63.3	62.4	62.5	63.8	64.7
	120	54.7	50.5	49.1	48.9	49.1	58.6	55.7	55.0	55.3	55.7		120	60.7	57.0	55.5	55.6	56.2	64.8	62.5	61.5	62.2	63.0
	100	56.4	51.2	49.0	48.2	48.1	60.0	56.1	54.7	54.4	54.5		100	63.0	57.7	55.1	54.6	54.9	67.1	63.2	61.3	61.3	61.7
		3	5	7	9	10	3	5	7	9	10			3	5	7	9	10	3	5	7	9	10
					Total/HDL cholesterol ratio												Total/HDL cholesterol ratio						

HDL, high-density lipoprotein.

### Demonstrating the CVD policy model

**Figure 4:**
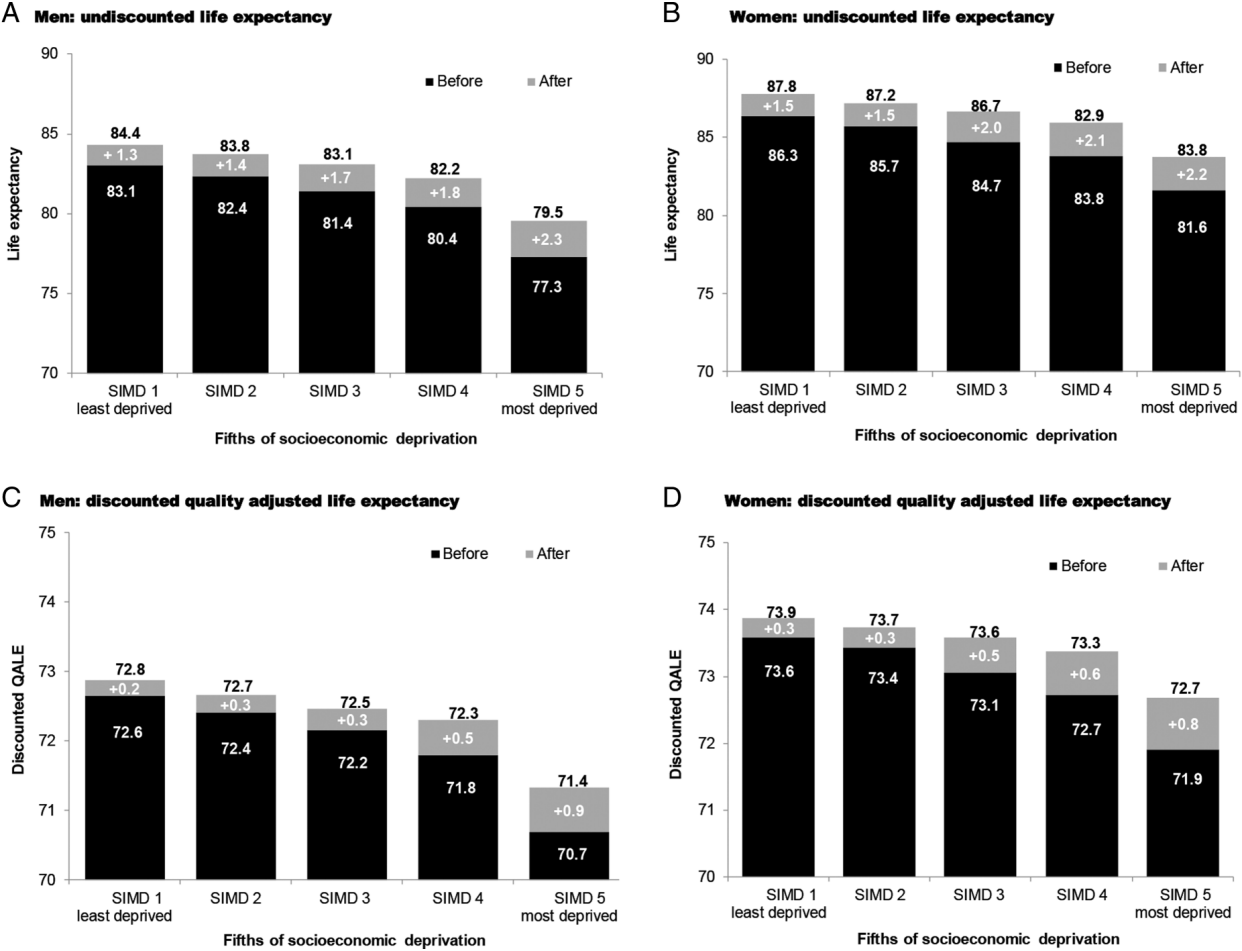
Risk factor modification on men and women aged 60 years—undiscounted life expectancy and discounted QALE. SIMD, Scottish Index of Multiple Deprivation; QALE, quality-adjusted life expectancy.

Figure 4 illustrates the potential gains from modifying the ‘average’ risk profiles of 60 year old men and women in the general population, across fifths of socioeconomic deprivation (SIMD), to the ‘ideal’ risk profiles according to clinical guidelines. Separate estimates are shown for undiscounted life expectancy and discounted QALE. For each column, the dark shading illustrates baseline life expectancy before risk factor modification, the light shading illustrates the potential gain from moving to a ‘perfect’ risk profile and revised estimates of undiscounted life expectancy and discounted QALE are provided at the top.

Potential gains from modifying risk factors increase with deprivation status. Women also tend to have greater potential to benefit, except within the most deprived fifth. Before risk factor modification, men in the least deprived fifth (SIMD 1) have a 7% higher life expectancy than the most deprived fifth (SIMD 5) and a 3.5% higher discounted QALE. Following risk factor modification, the gradient closes to 4.8% and 2.7%, respectively. For women, the least deprived fifth have 5.5% higher life expectancy than the most deprived fifth and 2.3% higher discounted QALE. Following risk factor modification, the gradient closes to 2.7% and 1.9%, respectively.

**Figure 5:**
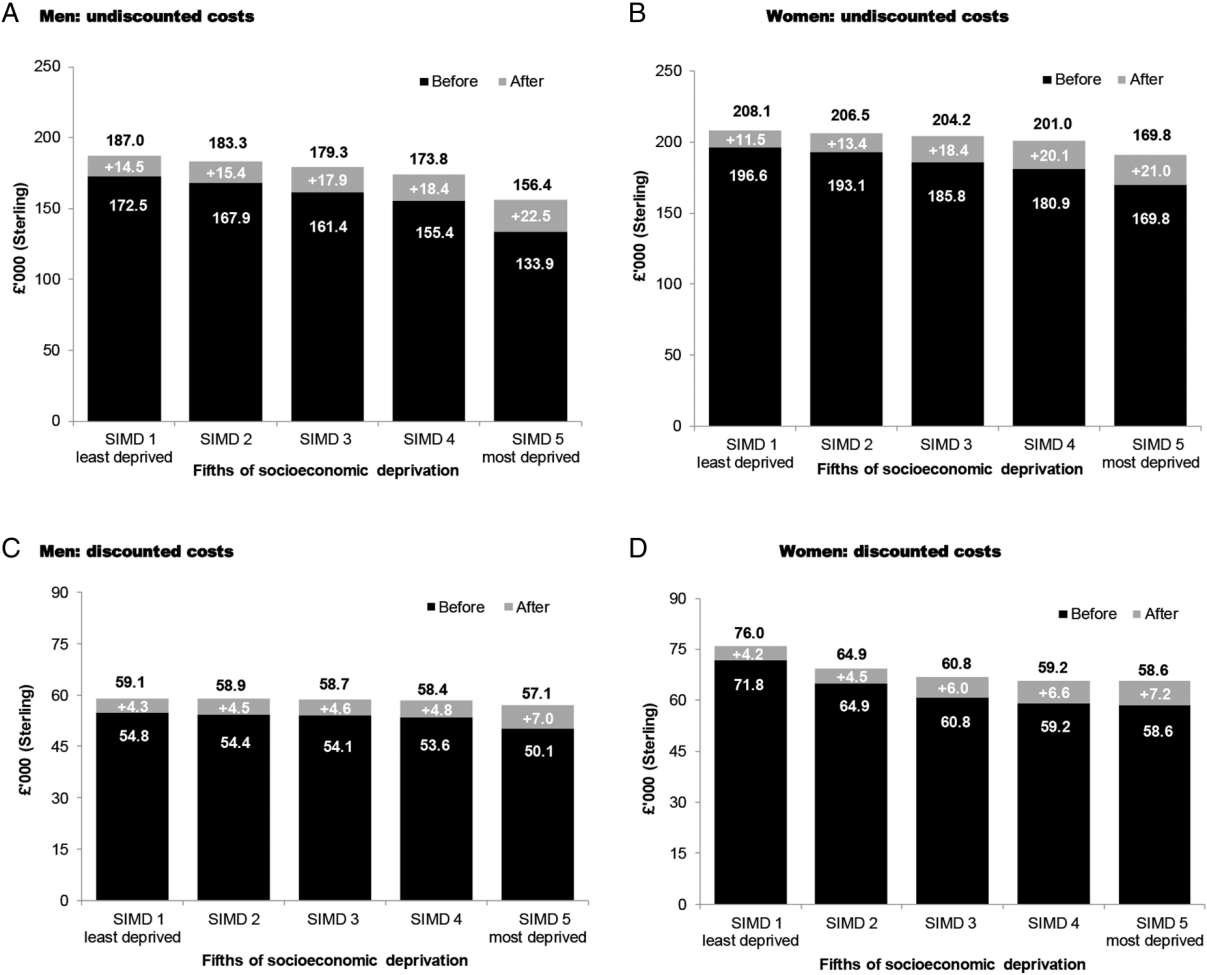
Risk factor modification on men and women aged 60 years—undiscounted and discounted lifetime hospital costs. SIMD, Scottish Index of Multiple Deprivation.

Figure 5 illustrates that individuals in the least deprived fifth (compared with the most deprived fifth) and women (compared with men) have higher costs consistent with longer life expectancies. Shifting individuals to a perfect risk profile results in increasing costs across all individuals given longer life expectancies. The effect of discounting is to give progressively less weight to the future and so narrows the gradient in lifetime costs between groups.

## Discussion

### Summary

We built a policy model capable of estimating life expectancy, QALE and lifetime hospital costs for individuals defined by the ASSIGN risk factors. The model can be used to estimate the cost-effectiveness of primary prevention interventions and assess impacts on health inequalities, which is the principal rationale of primary prevention in Scotland.^6^

### Strengths

A strength of the model is the ability to assess cost-effectiveness over the lifetime of individuals. This is in contrast, for instance, with the NICE Programme Development Group where the potential impacts of changes in legislation (eg, salt reduction) were projected over a period of 10 years.^22^

The model can be used to estimate the impact of a wide range of interventions aimed at reducing modifiable ASSIGN risk factors, including total cholesterol, HDL cholesterol, systolic blood pressure and cigarettes smoked per day. In this sense, the model may be described as a ‘generic model’, in contrast with ‘bespoke models’ built for specific interventions focused on particular risk factors. Further, the relationship between risk factors and events was estimated on a continuous basis where possible, enabling more fine-grained estimates compared with most other policy models that allocate individuals to subgroups (eg, a specified range of a risk factor).

The model can also be used to undertake ‘what-if’ analysis to assess the potential impact of individually targeted and population interventions, including ‘threshold analysis’ to vary the cost and/or effect of an intervention to assess the point at which an intervention becomes cost-effective.^20^

The policy model can be described as a state-transition model and took a competing risk approach (see the first paper) to estimate the relationship between risk factors and CVD and non-CVD death (eg, from cancer and respiratory disease). Consequently, the model can estimate the impact of risk factor modification beyond CVD events when conducting cost-effectiveness analysis.

The model could be used in practice to assess the cost-effectiveness of single interventions or multifactorial programmes, involving simultaneous interventions, that impact on the modifiable risk factors. For instance, Scotland's primary prevention programme, Keep Well, screens individuals using the ASSIGN risk tool with potential referral to a wide range of pharmaceutical and lifestyle interventions.^6^ At present, no economic evaluation has been undertaken, and there is an absence of robust economic evidence for multifactorial programmes in general.

A potentially important feature of the model is that by using the ASSIGN risk factor variables, which includes a measure of socioeconomic deprivation (SIMD), the model can take into account the impact of interventions on health inequalities. The model estimated the potential impacts if average modifiable risk profiles of men and women aged 60 years within SIMD fifths were switched to ‘perfect’ risk profiles. It was shown that primary prevention could potentially close health inequalities with more deprived groups having the most to gain. However, this exercise was for illustration to demonstrate the functionality of the model. Trial evidence is crucial, especially to understand reversibility of risks which may decrease with age, and long-term adherence/compliance which may be lower in socioeconomically disadvantaged groups. Trials and modelling can complement one another, with the latter projecting longer term clinical and economic outcomes where necessary.^23^

The model adjusts relatively comprehensively for morbidity impacts, including background morbidity and the impacts of experiencing first and subsequent non-fatal CVD events. Further, the model estimates the impact of extending life expectancy on hospitalisation costs. This is potentially important as prevention is likely to extend life expectancy and lead to individuals accumulating comorbidities.^24^ ^25^

A full uncertainty analysis, including PSA, can be undertaken. Further, the model is capable of undertaking decision analysis, including pretrial modelling, trial evaluation and value of information analysis.^3^ ^18^ ^20^ ^21^

Finally, a strength of the twinned papers and online supplementary appendix is the detailed reporting of data sources, methods, validation and calibration exercises. This practice follows recent modelling guidelines to enhance the transparency, peer review and use of models.^26^

### Limitations

There are several limitations to the model, in addition to those discussed in the first paper. A major limitation is that only hospitalisations are included when estimating health service costs. Not included, due to lack of data, are costs relating to primary care, prescriptions and community care. There is a need to consider linking primary and secondary healthcare data which are not routinely available across Scotland, at present. In evaluating primary prevention interventions, economic protocols should track all relevant costs in addition to intervention and hospitalisation cost(s), conditional on the perspective of the analysis. All such information could then be easily incorporated into the model to assess cost-effectiveness.

The utility decrements estimated for use in the policy model may not perfectly match the events incurred by individuals (eg, for CHD, the decrement for myocardial infarction is used). However, there is a lack of estimated utility decrements for the general population, none for Scotland, and these were the events considered within the SHeS.

Physical activity, which is an independent risk factor for CVD, is not part of the ASSIGN risk tool and therefore not part of the policy model. No other prominent CVD risk tool used routinely in clinical practice (that we are aware of) includes physical activity. Research has shown that there are diminishing marginal returns to adding risk factors,^27^ ASSIGN includes nine risk factors, and there is similar predictive ability between risk scores.^28^ To estimate the impact of physical activity interventions, the model could modify relevant ASSIGN risk factors (eg, systolic blood pressure, total cholesterol, HDL cholesterol) to match trial evidence concerning risk factors or event rates.

The model takes a ‘healthcare perspective’ with the focus on estimating the impact on (quality-adjusted) life expectancy to individuals and health service costs. This approach is consistent with guidance to undertake cost-effectiveness and cost-utility analysis.^1^ However, not considered at present are potential knock-on impacts of interventions on, for instance, carers and productivity.^19^

The model was developed using Scottish data sources and intended to be used in Scotland. However, the model could be used in other settings by recalibrating to the population of interest. For instance, the Framingham risk tool was developed in the USA but has been recalibrated to be used in England.^16^ Further, policy models such as the CHD Policy Model^29^ and IMPACT^30^ have been recalibrated to populations in different countries.

Overall, these limitations offer an opportunity for further research with guidance recommending that models be continuously improved, validated and calibrated to contemporary populations.^3^

### Policy applications

This CVD policy model is intended to inform primary prevention policy aimed at avoiding premature morbidity and mortality and associated health service costs. By using the same variables employed in the ASSIGN risk equation, there is alignment between the clinical tools currently used in Scotland to screen and prioritise individuals for intervention with this new policy model that can be used to evaluate the cost-effectiveness of individually targeted and population interventions aimed at modifying risk. Further, the model can assess impacts on health inequalities. Overall, the policy model attempts to integrate CVD risk estimation, individual patient decision-making and societal policymaking into a cohesive whole.
