# Supplementary material for: A cardiovascular disease policy model: part 2—preparing for economic evaluation and to assess health inequalities
Source: Open Heart. 2016 Jun 10;3(1):e000140. doi: 10.1136/openhrt-2014-000140 (PMC4908904; doi:10.1136/openhrt-2014-000140)
Supplement: Supplementary appendix [file openhrt-2014-000140supp.pdf]

On-line appendix: An alternative Cardiovascular Disease Policy Model

This appendix provides technical details of our model and is intended to be read in conjunction with the twinned papers[1-2].

Estimating risk of having first event (part 1 paper)

Gompertz regression survival analysis was used to model the cause specific hazards of the competing first events (non-fatal coronary heart disease (CHD), non-fatal cerebrovascular disease (CBVD), fatal CVD, fatal non-CVD). The model results are shown in Table A1. The model results are shown in Table A1. The associated Choleskey decomposition matrixes are shown in Tables A13-A20.

The following was derived from section 3.2 in Putter *et al.* using parametric expressions for the cause specific hazards[3]. The predicted cumulative incidence estimates of first events can be obtained from the Gompertz regression as follows:

$$\mathrm{CI}_{\mathrm{k}}\ (t) = \Sigma\ p_k(t_j)$$

i.e., the cumulative incidence at time *t*, $\mathrm{CI}_{\mathrm{k}}\ (t_j)$, is the cumulative sum of the unconditional probabilities of having event type *k* at time t_j, $\Sigma\ p_k(t_j)$, up to and including time *t*. The unconditional probabilities are obtained by:

$$p_k(t_j) = h_k(t_j)\mathrm{S}(t_{j-1})$$

Where $h_k(t_j)$ is the cause specific hazard for event type *k* which for the Gompertz regression has the expression:

$$h_k(t_j) = \exp(\mathrm{xb})\exp(\gamma t)$$

Where xb is the linear predictor from the regression and γ is the ancillary shape parameter estimated from the data. S(*t*) is the probability of surviving from any of the four events at time *t* and is obtained by:

$$\mathrm{S}(t) = \Pi(1 - \Sigma\ h_k(t_j))$$

Where Σ $h_k(t_j)$ is the sum of the four cause specific hazards at time t_j.

Figure 2a in the part 1 paper displays CI_k (t) and S(t) for a particular covariate profile. Figure 3b in the part 1 paper shows the $p_k(t_j)$ for the same covariate profile.

Estimating life expectancy following non-fatal CHD and CBVD events (part 1 paper)

Gompertz regression was also used to model the hazard of death following a first non-fatal event. The model results are shown in Table A2. The associated Choleskey decomposition matrixes are shown in Tables A21-A24. A predicted survival curve extrapolated until the probability of surviving beyond that time point was zero can be obtained for each covariate profile. Figures 2b-2d in the part 1 paper show predicted survival curves for a particular covariate profile with different ages at first event. The area under the survival curve was obtained by applying the trapezoidal rule with half cycle correction and this provided an estimate of remaining life expectancy.

Estimating background morbidity (part 2 paper)

Table A3 shows the mean HRQoL scores by age and socio-economic deprivation groups in the Scottish Health Survey (SHeS) 2003 data set. These scores are used to weight survival probabilities in all arms of the model (see Figure 1 in part 2 paper).

Estimating utility decrements (part 2 paper)

SHeS 2003 data was also used to estimate utility decrements that are associated with CHD, stroke, intermittent claudication and other CVD events. SHeS participants were asked if a doctor had diagnosed any of these events in the past. Table A4 shows the linear regression results from modelling HRQoL scores using the above events as covariates (and age). A history of heart failure (HF) was not part of the SHeS questionnaire and so a decrement for HF events was taken from the literature[4].

Estimating annual probability of incurring events that will have utility decrement applied (part 2 paper)

As part of the process for quality adjusting life years it was necessary to model the probability of non-fatal CVD events identified in the last section that occur after the first non-fatal events. Using the linked Scottish Heart Health Extended Cohort (SHHEC) and Scottish Morbidity Records (SMR) data set, the proportions of patients who had these events recorded in hospital discharge diagnosis fields for every year since the first event were calculated involving all

patients who were alive at the start of the given year. If a patient was alive in a given year but had no hospitalisation then a zero was added to the proportion calculation. To illustrate, Figure A1 shows for men the observed proportions of HF hospitalisations over time since first event.

Expected proportions of non-fatal CVD events have to be obtained beyond the observed follow-up period. A restricted cubic spline (RCS) function (with 3 knots based on Harrell's recommended percentiles[5]) was used to represent years since first event and included in a probit regression alongside age at first event, family history and socio-economic status. A RCS method was adopted as the shape of the function is less influenced at the edges of the data than say a fractional polynomial approach. This is particularly important in this setting where the time function is being extrapolated beyond the observed follow-up period.

Then, to calculate the expected probabilities of non-fatal CVD events of type *i* at each model cycle (year) *t*, p_{it}, the regression coefficients are transformed to the probability scale:

$$p_{it} = F(\mathrm{xb}_i(t))$$

where *F* is the cumulative standard Normal distribution and $\mathrm{xb}_i(t)$ is the linear predictor from the probit regression for event type *i* at time period *t*. The estimated coefficients from the regression models can be found in Table A5. The associated Choleskey decomposition matrixes are shown in Tables A25-A45.

Estimating quality adjustment (part 2 paper)

To quality adjust, the expected dis-utility was obtained using a Kaplan-Meier sample average (KMSA) approach which is essentially a sum over time of the probability of surviving post first event at a given time point multiplied by the mean dis-utility at that time point. For men this is given by:

$$\text{Expected dis-utility} = \Sigma\, S(t)[(0.043\times p_{i=CHD,t})+(0.092\times p_{i=Stroke,t})+(0.025\times p_{i=Int.\ Claud.,t}) +(0.043\times p_{i=Oth.\ heart\ cond.,t}) +(0.1\times p_{i=HF.,t})]$$

where S(*t*) is survival probability after first event, the values (0.043, 0.092, etc.) are the utility decrements (see Table A4) and $p_{i=<event>,t}$ are the probabilities of non-fatal CVD events (see last section).

Figure 2 in the part 2 paper illustrates the process of quality adjustment for a particular covariate profile after a non-fatal CHD event. The grey bars represent the mean dis-utility at each time point and the curve is the survival probability after non-fatal CHD event.

Estimating lifetime health service costs (part 2 paper)

Costs were modelled both pre- and post-first events. If a patient was alive in a given year but had no hospitalisation then a zero was added to the proportion calculation. To illustrate, Figures A2 and A3 show for men the observed costs over time before and after first event, respectively. As for non-fatal CVD events, expected costs have to be obtained beyond the observed follow-up period and therefore a RCS approach was again adopted. Costs were modelled using linear regression with spline functions representing time, age at survey (for pre-first event models), age at first event (for post-first event models), family history and socio-economic status. The estimated coefficients from the regression models can be found in Table A6. The associated Choleskey decomposition matrixes are shown in Tables A46- A57.

Expected costs are estimated using the KMSE approach as in the last section:

$$\text{Expected cost} = \Sigma\ S(t)xb(t))$$

For expected costs before first events, $S(t)$ is the probability of surviving from any of the four events at time t and $xb(t)$ is the linear predictor from the appropriate regression pre-first event model at time period t. For expected costs after first events, $S(t)$ is the probability of surviving post first event at time t and $xb(t)$ is the linear predictor from the appropriate regression post-first event model at time period t.

Figure 3 in the part 2 paper illustrates the process of estimating lifetime health service costs for a particular covariate profile after a non-fatal CHD event. The grey bars represent the mean costs at each time point and the curve is the survival probability after non-fatal CHD event.

Table A1: Gompertz regression modelling of cause specific hazards of first event

a) Men

Covariate	non-fatal CHD coeff. (95% CI)	p value	non-fatal CBVD coeff. (95% CI)	p value	fatal CVD coeff. (95% CI)	p value	fatal non-CVD coeff. (95% CI)	p value
Age	0.045 (0.038, 0.052)	<0.001	0.066 (0.054, 0.078)	<0.001	0.093 (0.082, 0.103)	<0.001	0.094 (0.085, 0.102)	<0.001
SIMD sc.	0.004 (0.001, 0.007)	0.002	0.009 (0.005, 0.014)	<0.001	0.006 (0.003, 0.010)	<0.001	0.009 (0.007, 0.012)	<0.001
Diabetes	0.653 (0.292, 1.014)	<0.001	1.168 (0.664, 1.673)	<0.001	0.863 (0.389, 1.337)	<0.001	0.335 (-0.169, 0.839)	0.192
Fam. his.	0.408 (0.295, 0.522)	<0.001	-0.021 (-0.235, 0.193)	0.847	0.165 (-0.003, 0.332)	0.054	-0.015 (-0.162, 0.133)	0.845
CPD	0.018 (0.013, 0.022)	<0.001	0.024 (0.017, 0.031)	<0.001	0.031 (0.026, 0.037)	<0.001	0.031 (0.026, 0.035)	<0.001
SBP	0.008 (0.005, 0.011)	<0.001	0.012 (0.007, 0.016)	<0.001	0.015 (0.012, 0.019)	<0.001	-0.001 (-0.005, 0.002)	0.391
TC	0.255 (0.208, 0.302)	<0.001	0.083 (-0.002, 0.168)	0.056	0.120 (0.050, 0.189)	0.001	-0.051 (-0.111, 0.008)	0.092
HDL	-0.760 (-0.947, -0.574)	<0.001	-0.125 (-0.394, 0.144)	0.362	-0.143 (-0.370, 0.085)	0.218	0.384 (0.214, 0.554)	<0.001
Constant	-9.54 (-10.12, -8.96)	<0.001	-12.51 (-13.47, -11.54)	<0.001	-14.13 (-14.93, -13.33)	<0.001	-11.22 (-11.91, -10.53)	<0.001
Gamma	0.057 (0.049, 0.065)	<0.001	0.091 (0.076, 0.105)	<0.001	0.079 (0.068, 0.091)	<0.001	0.081 (0.071, 0.091)	<0.001

b) Women

Covariate	non-fatal CHD coeff. (95% CI)	p value	non-fatal CBVD coeff. (95% CI)	p value	fatal CVD coeff. (95% CI)	p value	fatal non-CVD coeff. (95% CI)	p value
Age	0.058 (0.049, 0.067)	<0.001	0.080 (0.065, 0.095)	<0.001	0.102 (0.087, 0.116)	<0.001	0.091 (0.081, 0.101)	<0.001
SIMD sc.	0.009 (0.006, 0.012)	<0.001	0.013 (0.009, 0.018)	<0.001	0.004 (0.000, 0.009)	0.054	0.007 (0.004, 0.010)	<0.001
Diabetes	0.725 (0.343, 1.108)	<0.001	1.101 (0.595, 1.607)	<0.001	1.144 (0.680, 1.609)	<0.001	-0.037 (-0.668, 0.594)	0.908
Fam. his.	0.516 (0.389, 0.643)	<0.001	0.356 (0.150, 0.562)	0.001	0.239 (0.050, 0.428)	0.013	-0.018 (-0.165, 0.129)	0.814
CPD	0.021 (0.014, 0.027)	<0.001	0.027 (0.017, 0.037)	<0.001	0.048 (0.040, 0.056)	<0.001	0.038 (0.032, 0.044)	<0.001
SBP	0.006 (0.003, 0.009)	<0.001	0.014 (0.009, 0.018)	<0.001	0.018 (0.013, 0.022)	<0.001	0.003 (-0.001, 0.006)	0.112
TC	0.188 (0.136, 0.240)	<0.001	-0.051 (-0.150, 0.048)	0.308	0.057 (-0.022, 0.136)	0.159	-0.076 (-0.140, -0.013)	0.018
HDL	-0.746 (-0.937, -0.555)	<0.001	-0.346 (-0.636, -0.056)	0.020	-0.174 (-0.420, 0.073)	0.166	-0.045 (-0.229, 0.138)	0.626
Constant	-10.52 (-11.14, -9.89)	<0.001	-13.01 (-14.02, -12.00)	<0.001	-15.42 (-16.39, -14.46)	<0.001	-11.26 (-11.93, -10.58)	<0.001
Gamma	0.083 (0.073, 0.093)	<0.001	0.096 (0.080, 0.112)	<0.001	0.099 (0.084, 0.114)	<0.001	0.089 (0.078, 0.099)	<0.001

Note: ‘Age’ is age at survey

Table A2: Gompertz regression modelling of survival after non-fatal event

a) Men, after CHD event

Covariate	coeff. (95% CI)	p value
Age at event	0.077 (0.065, 0.090)	<0.001
SIMD score	0.013 (0.009, 0.017)	<0.001
Family history	-0.035 (-0.233, 0.162)	0.725
Constant	-8.597 (-9.479, -7.715)	<0.001
Gamma	0.038 (0.017, 0.059)	<0.001

b) Men, after CBVD event

Covariate	coeff. (95% CI)	p value
Age at event	0.067 (0.048, 0.087)	<0.001
SIMD score	0.009 (0.003, 0.015)	0.004
Family history	0.061 (-0.263, 0.385)	0.711
Constant	-7.447 (-8.852, -6.043)	<0.001
Gamma	0.024 (-0.015, 0.062)	0.226

c) Women, after CHD event

Covariate	coeff. (95% CI)	p value
Age at event	0.074 (0.059, 0.089)	<0.001
SIMD score	0.007 (0.003, 0.012)	0.003
Family history	-0.285 (-0.519, -0.051)	0.017
Constant	-8.360 (-9.488, -7.232)	<0.001
Gamma	0.041 (0.013, 0.068)	0.004

d) Women, after CBVD event

Covariate	coeff. (95% CI)	p value
Age at event	0.070 (0.050, 0.089)	<0.001
SIMD score	0.0002 (-0.007, 0.008)	0.961
Family history	0.181 (-0.152, 0.513)	0.287
Constant	-7.668 (-9.192, -6.144)	<0.001
Gamma	0.036 (-0.008, 0.080)	0.106

Table A3: Mean (and 95% CI) HRQoL scores in the general Scottish population by age group and fifths of SIMD group

a) Men

Age group (years)	SIMD 1 least deprived	SIMD 2	SIMD 3	SIMD 4	SIMD 5 most deprived
<25	0.822 (0.787, 0.857)	0.853 (0.824, 0.881)	0.834 (0.795, 0.873)	0.857 (0.828, 0.887)	0.787 (0.745, 0.830)
25-34	0.848 (0.826, 0.871)	0.849 (0.830, 0.867)	0.823 (0.796, 0.850)	0.816 (0.794, 0.839)	0.774 (0.741, 0.807)
35-44	0.834 (0.816, 0.852)	0.838 (0.820, 0.855)	0.823 (0.805, 0.841)	0.811 (0.787, 0.835)	0.777 (0.744, 0.810)
45-54	0.825 (0.805, 0.845)	0.827 (0.806, 0.849)	0.808 (0.783, 0.833)	0.791 (0.756, 0.826)	0.762 (0.729, 0.794)
55-64	0.845 (0.826, 0.865)	0.803 (0.780, 0.826)	0.820 (0.796, 0.843)	0.782 (0.751, 0.813)	0.718 (0.680, 0.757)
65-74	0.813 (0.784, 0.841)	0.822 (0.791, 0.853)	0.802 (0.775, 0.830)	0.761 (0.729, 0.792)	0.732 (0.697, 0.768)
75+	0.797 (0.750, 0.843)	0.802 (0.770, 0.835)	0.756 (0.722, 0.791)	0.775 (0.731, 0.818)	0.732 (0.685, 0.779)

b) Women

Age group (years)	SIMD 1 least deprived	SIMD 2	SIMD 3	SIMD 4	SIMD 5 most deprived
<25	0.846 (0.813, 0.880)	0.810 (0.779, 0.842)	0.780 (0.739, 0.821)	0.799 (0.765, 0.832)	0.816 (0.787, 0.845)
25-34	0.839 (0.822, 0.856)	0.816 (0.795, 0.836)	0.822 (0.803, 0.841)	0.803 (0.780, 0.825)	0.775 (0.749, 0.800)
35-44	0.837 (0.823, 0.852)	0.827 (0.812, 0.841)	0.794 (0.773, 0.815)	0.788 (0.769, 0.808)	0.748 (0.722, 0.774)
45-54	0.827 (0.812, 0.843)	0.793 (0.773, 0.812)	0.780 (0.758, 0.802)	0.769 (0.745, 0.792)	0.736 (0.708, 0.764)
55-64	0.835 (0.816, 0.854)	0.815 (0.798, 0.832)	0.791 (0.768, 0.814)	0.769 (0.742, 0.796)	0.701 (0.670, 0.732)
65-74	0.827 (0.803, 0.851)	0.803 (0.776, 0.830)	0.792 (0.766, 0.818)	0.742 (0.709, 0.776)	0.702 (0.668, 0.736)
75+	0.741 (0.702, 0.779)	0.765 (0.732, 0.798)	0.715 (0.681, 0.748)	0.693 (0.655, 0.731)	0.689 (0.652, 0.726)

Table A4: Event utility decrements

a) Men

Covariate	coeff. (95% CI)	p value
CHD	0.043 (0.019, 0.068)	0.001
Stroke	0.092 (0.061, 0.122)	<0.001
Intermittent claudication	0.025 (-0.005, 0.056)	0.101
Other heart condition	0.043 (0.011, 0.074)	0.008

b) Women

Covariate	coeff. (95% CI)	p value
CHD	0.037 (0.007, 0.067)	0.016
Stroke	0.097 (0.067, 0.127)	<0.001
Intermittent claudication	0.017 (-0.009, 0.043)	0.211
Other heart condition	0.023 (-0.011, 0.058)	0.188

Table A5: Modelling the probability of non-fatal CVD events after first non-fatal event

a) Men, after CHD event

Covariate	CHD		Stroke		Inter. claudication		Other heart condition	
	coeff. (95% CI)	p value	coeff. (95% CI)	p value	coeff. (95% CI)	p value	coeff. (95% CI)	p value
ti1	-0.019 (-0.040, 0.002)	0.073	-0.101 (-0.147, -0.055)	<0.001	-0.049 (-0.104, 0.006)	0.080	-0.060 (-0.091, -0.028)	<0.001
ti2	0.071 (0.045, 0.098)	<0.001	0.098 (0.035, 0.161)	0.002	0.034 (-0.039, 0.107)	0.363	0.050 (0.008, 0.092)	0.019
Age	0.010 (0.005, 0.016)	<0.001	0.001 (-0.007, 0.009)	0.821	-0.001 (-0.012, 0.010)	0.888	0.005 (-0.002, 0.013)	0.150
SIMD sc.	0.003 (0.001, 0.005)	0.004	0.003 (0.000, 0.007)	0.051	0.002 (-0.003, 0.006)	0.444	0.003 (0.001, 0.006)	0.012
Fam. his.	0.106 (0.019, 0.193)	0.016	-0.011 (-0.168, 0.145)	0.886	-0.136 (-0.350, 0.077)	0.211	0.174 (0.051, 0.297)	0.006
Constant	-2.012 (-2.420, -1.605)	<0.001	-2.085 (-2.640, -1.530)	<0.001	-2.137 (-2.903, -1.371)	<0.001	-2.187 (-2.680, -1.693)	<0.001

Covariate	HF	
	coeff. (95% CI)	p value
ti1	-0.152 (-0.195, -0.110)	<0.001
ti2	0.160 (0.098, 0.223)	<0.001
Age	0.014 (0.004, 0.023)	0.004
SIMD sc.	0.002 (-0.002, 0.006)	0.297
Fam. his.	0.044 (-0.121, 0.210)	0.599
Constant	-2.626 (-3.260, -1.992)	<0.001

b) Men, after CBVD event

Covariate	CHD coeff. (95% CI)	p value	Stroke coeff. (95% CI)	p value	Inter. claudication coeff. (95% CI)	p value	Other heart condition coeff. (95% CI)	p value
ti1	-0.069 (-0.150, 0.012)	0.095	-0.035 (-0.087, 0.017)	0.183	0.020 (-0.085, 0.125)	0.712	-0.070 (-0.134, -0.006)	0.032
ti2	0.063 (-0.049, 0.174)	0.270	0.046 (-0.030, 0.123)	0.235	-0.108 (-0.305, 0.090)	0.285	0.077 (-0.016, 0.170)	0.103
Age	-0.003 (-0.016, 0.010)	0.613	0.010 (-0.001, 0.021)	0.075	0.001 (-0.016, 0.019)	0.873	0.004 (-0.009, 0.017)	0.537
SIMD sc.	-0.002 (-0.008, 0.004)	0.455	0.003 (-0.000, 0.006)	0.073	0.007 (0.001, 0.014)	0.030	0.002 (-0.002, 0.007)	0.315
Fam. his.	0.144 (-0.085, 0.373)	0.218	0.019 (-0.154, 0.191)	0.833	0.024 (-0.340, 0.389)	0.895	0.053 (0.169, 0.275)	0.641
Constant	-1.506 (-2.420, -1.605)	0.001	-2.109 (-2.891, -1.327)	<0.001	-2.744 (-4.034, -1.454)	<0.001	-1.923 (-2.789, -1.058)	<0.001

Covariate	HF coeff. (95% CI)	p value
ti1	-0.129 (-0.275, 0.017)	0.083
ti2	0.159 (-0.021, 0.340)	0.084
Age	0.039 (0.009, 0.070)	0.012
SIMD sc.	-0.010 (-0.021, 0.001)	0.079
Fam. his.	0.353 (-0.063, 0.770)	0.097
Constant	-4.697 (-6.983, -2.410)	<0.001

c) Women, after CHD event

Covariate	CHD coeff. (95% CI)	p value	Stroke coeff. (95% CI)	p value	Inter. claudication coeff. (95% CI)	p value	Other heart condition coeff. (95% CI)	p value
ti1	-0.003 (-0.033, 0.027)	0.823	-0.072 (-0.144, 0.000)	0.051	-0.078 (-0.152, -0.004)	0.040	-0.045 (-0.087, -0.003)	0.037
ti2	0.057 (0.011, 0.103)	0.016	0.026 (-0.102, 0.155)	0.688	0.106 (-0.015, 0.228)	0.086	0.026 (-0.045, 0.096)	0.477
Age	0.010 (0.004, 0.016)	0.002	0.007 (-0.006, 0.019)	0.281	0.016 (-0.001, 0.033)	0.061	0.012 (0.003, 0.021)	0.009
SIMD sc.	0.001 (-0.001, 0.004)	0.198	0.005 (0.001, 0.009)	0.014	0.001 (-0.005, 0.008)	0.705	0.001 (-0.002, 0.004)	0.484
Fam. his.	0.056 (-0.046, 0.158)	0.279	-0.014 (-0.221, 0.194)	0.898	-0.129 (-0.379, 0.122)	0.314	-0.138 (-0.270, -0.005)	0.041
Constant	-2.137 (-2.580, -1.694)	<0.001	-2.638 (-3.492, -1.783)	<0.001	-3.274 (-4.558, -1.990)	<0.001	-2.407 (-3.00, -1.810)	<0.001

Covariate	HF coeff. (95% CI)	p value
ti1	-0.119 (-0.176, -0.061)	<0.001
ti2	0.133 (0.039, 0.227)	0.005
Age	0.018 (0.00, 0.029)	0.003
SIMD sc.	0.004 (-0.000, 0.009)	0.077
Fam. his.	-0.038 (-0.246, 0.170)	0.721
Constant	-3.017 (-3.857, -2.177)	<0.001

d) Women, after CBVD event

Covariate	CHD		Stroke		Inter. claudication		Other heart condition	
	coeff. (95% CI)	p value	coeff. (95% CI)	p value	coeff. (95% CI)	p value	coeff. (95% CI)	p value
ti1	0.078 (-0.028, 0.183)	0.150	-0.023 (-0.077, 0.030)	0.392	0.008 (-0.137, 0.152)	0.916	-0.053 (-0.138, 0.032)	0.222
ti2	-0.088 (-0.225, 0.049)	0.209	0.056 (-0.020, 0.132)	0.150	-0.069 (-0.275, 0.137)	0.513	0.034 (-0.071, 0.140)	0.524
Age	-0.0004 (-0.014, 0.013)	0.951	0.022 (0.013, 0.030)	<0.001	-0.011 (-0.031, 0.009)	0.279	0.006 (-0.005, 0.017)	0.302
SIMD sc.	0.004 (-0.003, 0.011)	0.304	0.001 (-0.002, 0.004)	0.525	0.0004 (-0.013, 0.014)	0.955	-0.001 (-0.006, 0.004)	0.749
Fam. his.	0.068 (-0.222, 0.359)	0.644	0.042 (-0.192, 0.108)	0.582	-0.303 (-0.798, 0.192)	0.230	0.281 (0.044, 0.517)	0.020
Constant	-2.531 (-3.653, -1.409)	<0.001	-2.777 (-3.424, -2.130)	<0.001	-1.714 (-3.543, 0.115)	0.066	-2.178 (-2.983, -1.373)	<0.001

Covariate	HF	
	coeff. (95% CI)	p value
ti1	-0.186 (-0.319, -0.054)	0.006
ti2	0.227 (0.069, 0.386)	0.005
Age	-0.002 (-0.017, 0.014)	0.839
SIMD sc.	0.001 (-0.007, 0.010)	0.757
Fam. his.	0.036 (-0.360, 0.432)	0.859
Constant	-1.881 (-3.219, -0.542)	0.006

Note: ‘Age’ is age at first event; ‘ti1’ and ‘ti2’ are time spline function variables (see Tables A7-A10)

Table A6: Modelling mean costs pre- and post-first events

a) Men, pre-first event

Covariate	non-fatal CHD		non-fatal CBVD		fatal CVD		fatal non-CVD	
	coeff. (95% CI)	p value	coeff. (95% CI)	p value	coeff. (95% CI)	p value	coeff. (95% CI)	p value
ti1	18.6 (-2.0, 39.2)	0.077	5.5 (-38.0, 49.0)	0.804	26.1 (-7.1, 59.3)	0.123	42.1 (-4.5, 88.7)	0.076
ti2	115.0 (70.3, 159.8)	<0.001	156.6 (72.0, 241.2)	<0.001	114.6 (56.8, 172.5)	<0.001	237.2 (157.1, 317.4)	<0.001
Age	22.7 (16.4, 29.0)	<0.001	17.3 (5.8, 28.9)	0.003	27.7 (16.5, 38.89)	<0.001	24.7 (9.0, 40.5)	0.002
SIMD sc.	5.2 (3.0, 7.4)	<0.001	6.6 (2.9, 10.3)	<0.001	3.8 (-0.1, 7.8)	0.059	4.7 (-0.5, 10.0)	0.078
Fam. his.	93.8 (1.5, 186.1)	0.046	-161.9 (-328.5, 4.8)	0.057	67.4 (-120.9, 255.7)	0.483	116.9 (-198.7, 432.6)	0.468
Constant	-1121 (-1446, -795)	<0.001	-832.8 (-1483, -182.4)	0.012	-1345 (-1981, -709.2)	<0.001	-1029 (-1890, -169.2)	0.019

b) Women, pre-first event

Covariate	non-fatal CHD		non-fatal CBVD		fatal CVD		fatal non-CVD	
	coeff. (95% CI)	p value	coeff. (95% CI)	p value	coeff. (95% CI)	p value	coeff. (95% CI)	p value
ti1	-7.3 (-94.3, 79.8)	0.870	14.2 (-23.3, 51.6)	0.458	23.9 (-18.4, 66.2)	0.269	59.0 (11.5, 106.4)	0.015
ti2	172.2 (-64.6, 408.9)	0.154	121.8 (57.7, 185.8)	<0.001	144.7 (72.2, 217.3)	<0.001	202.6 (125.8, 279.4)	<0.001
Age	0.5 (-25.1, 26.2)	0.967	26.3 (15.0, 37.6)	<0.001	33.7 (18.4, 49.0)	<0.001	16.0 (-3.5, 35.4)	0.107
SIMD sc.	10.6 (2.3, 19.0)	0.013	8.4 (2.4, 14.4)	0.006	5.5 (0.8, 10.2)	0.022	11.9 (5.6, 18.3)	<0.001
Fam. his.	337.8 (-235.7, 911.4)	0.248	22.7 (-176.6, 222.0)	0.823	105.5 (-125.0, 336.0)	0.370	47.7 (-229.7, 325.2)	0.736
Constant	-214.2 (-1359, 930.8)	0.714	-1462 (-2123, -800.5)	<0.001	-1727 (-2643, -809.9)	<0.001	-832.0 (-1894, 230.1)	0.125

Note: ‘Age’ is age at survey; ‘ti1’ and ‘ti2’ are time spline function variables (see Tables A11-A12)

c) Men, after non-fatal CHD event

Covariate	coeff. (95% CI)	p value
ti1	-552.6 (-638.7, -466.6)	<0.001
ti2	654.9 (554.5, 755.3)	<0.001
Age at event	84.6 (66.9, 102.4)	<0.001
SIMD score	14.2 (7.6, 20.8)	<0.001
Family history	239.8 (-80.4, 560.0)	0.142
Constant	-1024 (-2107, 59.0)	<0.001

d) Men, after non-fatal CBVD event

Covariate	coeff. (95% CI)	p value
ti1	-680.0 (-854.7, -505.2)	<0.001
ti2	787.7 (555.7, 1020)	<0.001
Age at event	112.6 (81.2, 144.0)	<0.001
SIMD score	6.8 (-4.5, 18.1)	0.236
Family history	-102.2 (-717.2, 512.9)	0.745
Constant	-1836 (-4010, 338.3)	0.098

e) Women, after non-fatal CHD event

Covariate	coeff. (95% CI)	p value
ti1	-548.6 (-652.4, -444.8)	<0.001
ti2	745.4 (600.4, 890.3)	<0.001
Age at event	90.7 (68.5, 112.9)	<0.001
SIMD score	13.6 (6.0, 21.3)	<0.001
Family history	-227.9 (-596.5, 140.7)	0.226
Constant	-1321 (-2900, 257.1)	<0.001

f) Women, after non-fatal CBVD event

Covariate	coeff. (95% CI)	p value
ti1	-542.3 (-744.1, -340.4)	<0.001
ti2	595.6 (357.4, 833.9)	<0.001
Age at event	97.1 (67.0, 127.2)	<0.001
SIMD score	7.7 (-4.7, 20.0)	0.223
Family history	-93.9 (-656.1, 468.4)	0.743
Constant	-1251 (-3593, 1092)	0.295

Note: ‘ti1’ and ‘ti2’ are time spline function variables (see Tables A7-A10)

Table A7: Time spline variables for models in Tables A5a and A6c

year	ti1	ti2
1	1	0
2	2	0.005917
3	3	0.047337
4	4	0.159763
5	5	0.378698
6	6	0.739645
7	7	1.268491
8	8	1.952663
9	9	2.76997
10	10	3.698225
11	11	4.715237
12	12	5.798817
13	13	6.926775
14	14	8.076923
15	15	9.230769
16	16	10.38461
17	17	11.53846
18	18	12.69231
19	19	13.84615
20	20	15
21	21	16.15385
22	22	17.30769
23	23	18.46154
24	24	19.61539
25	25	20.76923
26	26	21.92308
27	27	23.07692
28	28	24.23077
29	29	25.38461
30	30	26.53846
31	31	27.69231
32	32	28.84615
33	33	30
34	34	31.15385
35	35	32.30769
36	36	33.46154
37	37	34.61538
38	38	35.76923
39	39	36.92308
40	40	38.07692
41	41	39.23077
42	42	40.38462
43	43	41.53846
44	44	42.69231
45	45	43.84615
46	46	45
47	47	46.15385
48	48	47.30769
49	49	48.46154
50	50	49.61538
51	51	50.76923
52	52	51.92308
53	53	53.07692
54	54	54.23077
55	55	55.38462
56	56	56.53846
57	57	57.69231
58	58	58.84615
59	59	60
60	60	61.15385
61	61	62.30769
62	62	63.46154
63	63	64.61539
64	64	65.76923
65	65	66.92308
66	66	68.07692
67	67	69.23077
68	68	70.38461
69	69	71.53846
70	70	72.69231
71	71	73.84615
72	72	75
73	73	76.15385
74	74	77.30769
75	75	78.46154
76	76	79.61539
77	77	80.76923
78	78	81.92308
79	79	83.07692
80	80	84.23077
81	81	85.38461
82	82	86.53846
83	83	87.69231
84	84	88.84615
85	85	90
86	86	91.15385
87	87	92.30769
88	88	93.46154
89	89	94.61539
90	90	95.76923
91	91	96.92308
92	92	98.07692
93	93	99.23077
94	94	100.3846
95	95	101.5385
96	96	102.6923
97	97	103.8462
98	98	105
99	99	106.1538
100	100	107.3077

Table A8: Time spline variables for models in Tables A5b and A6d

year	ti1	ti2
1	1	0
2	2	0.008265
3	3	0.066116
4	4	0.223141
5	5	0.528926
6	6	1.020071
7	7	1.681228
8	8	2.484061
9	9	3.400236
10	10	4.401417
11	11	5.459268
12	12	6.545455
13	13	7.636364
14	14	8.727273
15	15	9.818182
16	16	10.90909
17	17	12
18	18	13.09091
19	19	14.18182
20	20	15.27273
21	21	16.36364
22	22	17.45455
23	23	18.54545
24	24	19.63636
25	25	20.72727
26	26	21.81818
27	27	22.90909
28	28	24
29	29	25.09091
30	30	26.18182
31	31	27.27273
32	32	28.36364
33	33	29.45455
34	34	30.54545
35	35	31.63636
36	36	32.72727
37	37	33.81818
38	38	34.90909
39	39	36
40	40	37.09091
41	41	38.18182
42	42	39.27273
43	43	40.36364
44	44	41.45454
45	45	42.54546
46	46	43.63636
47	47	44.72727
48	48	45.81818
49	49	46.90909
50	50	48
51	51	49.09091
52	52	50.18182
53	53	51.27273
54	54	52.36364
55	55	53.45454
56	56	54.54546
57	57	55.63636
58	58	56.72727
59	59	57.81818
60	60	58.90909
61	61	60
62	62	61.09091
63	63	62.18182
64	64	63.27273
65	65	64.36364
66	66	65.45454
67	67	66.54546
68	68	67.63636
69	69	68.72727
70	70	69.81818
71	71	70.90909
72	72	72
73	73	73.09091
74	74	74.18182
75	75	75.27273
76	76	76.36364
77	77	77.45454
78	78	78.54546
79	79	79.63636
80	80	80.72727
81	81	81.81818
82	82	82.90909
83	83	84
84	84	85.09091
85	85	86.18182
86	86	87.27273
87	87	88.36364
88	88	89.45454
89	89	90.54546
90	90	91.63636
91	91	92.72727
92	92	93.81818
93	93	94.90909
94	94	96
95	95	97.09091
96	96	98.18182
97	97	99.27273
98	98	100.3636
99	99	101.4545
100	100	102.5455

Table A9: Time spline variables for models in Tables A5c and A6e

year	ti1	ti2	year	ti1	ti2	year	ti1	ti2
1	1	0	35	35	26.15385	68	68	56.61538
2	2	0.005917	36	36	27.07692	69	69	57.53846
3	3	0.047337	37	37	28	70	70	58.46154
4	4	0.159763	38	38	28.92308	71	71	59.38462
5	5	0.378698	39	39	29.84615	72	72	60.30769
6	6	0.731098	40	40	30.76923	73	73	61.23077
7	7	1.20973	41	41	31.69231	74	74	62.15385
8	8	1.798817	42	42	32.61538	75	75	63.07692
9	9	2.482577	43	43	33.53846	76	76	64
10	10	3.245233	44	44	34.46154	77	77	64.92308
11	11	4.071006	45	45	35.38462	78	78	65.84615
12	12	4.944116	46	46	36.30769	79	79	66.76923
13	13	5.848783	47	47	37.23077	80	80	67.69231
14	14	6.769231	48	48	38.15385	81	81	68.61539
15	15	7.692307	49	49	39.07692	82	82	69.53846
16	16	8.615385	50	50	40	83	83	70.46154
17	17	9.538462	51	51	40.92308	84	84	71.38461
18	18	10.46154	52	52	41.84615	85	85	72.30769
19	19	11.38461	53	53	42.76923	86	86	73.23077
20	20	12.30769	54	54	43.69231	87	87	74.15385
21	21	13.23077	55	55	44.61538	88	88	75.07692
22	22	14.15385	56	56	45.53846	89	89	76
23	23	15.07692	57	57	46.46154	90	90	76.92308
24	24	16	58	58	47.38462	91	91	77.84615
25	25	16.92308	59	59	48.30769	92	92	78.76923
26	26	17.84615	60	60	49.23077	93	93	79.69231
27	27	18.76923	61	61	50.15385	94	94	80.61539
28	28	19.69231	62	62	51.07692	95	95	81.53846
29	29	20.61539	63	63	52	96	96	82.46154
30	30	21.53846	64	64	52.92308	97	97	83.38461
31	31	22.46154	65	65	53.84615	98	98	84.30769
32	32	23.38461	66	66	54.76923	99	99	85.23077
33	33	24.30769	67	67	55.69231	100	100	86.15385
34	34	25.23077						

Table A10: Time spline variables for models in Tables A5d and A6f

year	ti1	ti2	year	ti1	ti2	year	ti1	ti2
1	1	0	35	35	31.63636	68	68	67.63636
2	2	0.008265	36	36	32.72727	69	69	68.72727
3	3	0.066116	37	37	33.81818	70	70	69.81818
4	4	0.223141	38	38	34.90909	71	71	70.90909
5	5	0.528926	39	39	36	72	72	72
6	6	1.020071	40	40	37.09091	73	73	73.09091
7	7	1.681228	41	41	38.18182	74	74	74.18182
8	8	2.484061	42	42	39.27273	75	75	75.27273
9	9	3.400236	43	43	40.36364	76	76	76.36364
10	10	4.401417	44	44	41.45454	77	77	77.45454
11	11	5.459268	45	45	42.54546	78	78	78.54546
12	12	6.545455	46	46	43.63636	79	79	79.63636
13	13	7.636364	47	47	44.72727	80	80	80.72727
14	14	8.727273	48	48	45.81818	81	81	81.81818
15	15	9.818182	49	49	46.90909	82	82	82.90909
16	16	10.90909	50	50	48	83	83	84
17	17	12	51	51	49.09091	84	84	85.09091
18	18	13.09091	52	52	50.18182	85	85	86.18182
19	19	14.18182	53	53	51.27273	86	86	87.27273
20	20	15.27273	54	54	52.36364	87	87	88.36364
21	21	16.36364	55	55	53.45454	88	88	89.45454
22	22	17.45455	56	56	54.54546	89	89	90.54546
23	23	18.54545	57	57	55.63636	90	90	91.63636
24	24	19.63636	58	58	56.72727	91	91	92.72727
25	25	20.72727	59	59	57.81818	92	92	93.81818
26	26	21.81818	60	60	58.90909	93	93	94.90909
27	27	22.90909	61	61	60	94	94	96
28	28	24	62	62	61.09091	95	95	97.09091
29	29	25.09091	63	63	62.18182	96	96	98.18182
30	30	26.18182	64	64	63.27273	97	97	99.27273
31	31	27.27273	65	65	64.36364	98	98	100.3636
32	32	28.36364	66	66	65.45454	99	99	101.4545
33	33	29.45455	67	67	66.54546	100	100	102.5455
34	34	30.54545						

Table A11: Time spline variables for models in Table A6a (non-fatal CHD)

year	ti1	ti2
1	1	0
2	2	0
3	3	0.004444
4	4	0.035556
5	5	0.12
6	6	0.284445
7	7	0.555556
8	8	0.953333
9	9	1.471111
10	10	2.095556
11	11	2.813333
12	12	3.611111
13	13	4.475555
14	14	5.393333
15	15	6.351111
16	16	7.335556
17	17	8.333333
18	18	9.333333
19	19	10.33333
20	20	11.33333
21	21	12.33333
22	22	13.33333
23	23	14.33333
24	24	15.33333
25	25	16.33333
26	26	17.33333
27	27	18.33333
28	28	19.33333
29	29	20.33333
30	30	21.33333
31	31	22.33333
32	32	23.33333
33	33	24.33333
34	34	25.33333
35	35	26.33333
36	36	27.33333
37	37	28.33333
38	38	29.33333
39	39	30.33333
40	40	31.33333
41	41	32.33333
42	42	33.33333
43	43	34.33333
44	44	35.33333
45	45	36.33333
46	46	37.33333
47	47	38.33333
48	48	39.33333
49	49	40.33333
50	50	41.33333
51	51	42.33333
52	52	43.33333
53	53	44.33333
54	54	45.33333
55	55	46.33333
56	56	47.33333
57	57	48.33333
58	58	49.33333
59	59	50.33333
60	60	51.33333
61	61	52.33333
62	62	53.33333
63	63	54.33333
64	64	55.33333
65	65	56.33333
66	66	57.33333
67	67	58.33333
68	68	59.33333
69	69	60.33333
70	70	61.33333
71	71	62.33333
72	72	63.33333
73	73	64.33334
74	74	65.33334
75	75	66.33334
76	76	67.33334
77	77	68.33334
78	78	69.33334
79	79	70.33334
80	80	71.33334
81	81	72.33334
82	82	73.33334
83	83	74.33334
84	84	75.33334
85	85	76.33334
86	86	77.33334
87	87	78.33334
88	88	79.33334
89	89	80.33334
90	90	81.33334
91	91	82.33334
92	92	83.33334
93	93	84.33334
94	94	85.33334
95	95	86.33334
96	96	87.33334
97	97	88.33334
98	98	89.33334
99	99	90.33334
100	100	91.33334

Table A12: Time spline variables for models in Table A6a (non-fatal CBVD, fatal CVD, fatal non-CVD) and A6b

year	ti1	ti2
1	1	0
2	2	0
3	3	0.004444
4	4	0.035556
5	5	0.12
6	6	0.284445
7	7	0.555556
8	8	0.96
9	9	1.517037
10	10	2.216296
11	11	3.04
12	12	3.97037
13	13	4.98963
14	14	6.08
15	15	7.223704
16	16	8.402963
17	17	9.6
18	18	10.8
19	19	12
20	20	13.2
21	21	14.4
22	22	15.6
23	23	16.8
24	24	18
25	25	19.2
26	26	20.4
27	27	21.6
28	28	22.8
29	29	24
30	30	25.2
31	31	26.4
32	32	27.6
33	33	28.8
34	34	30
35	35	31.2
36	36	32.4
37	37	33.6
38	38	34.8
39	39	36
40	40	37.2
41	41	38.4
42	42	39.6
43	43	40.8
44	44	42
45	45	43.2
46	46	44.4
47	47	45.6
48	48	46.8
49	49	48
50	50	49.2
51	51	50.4
52	52	51.6
53	53	52.8
54	54	54
55	55	55.2
56	56	56.4
57	57	57.6
58	58	58.8
59	59	60
60	60	61.2
61	61	62.4
62	62	63.6
63	63	64.8
64	64	66
65	65	67.2
66	66	68.4
67	67	69.6
68	68	70.8
69	69	72
70	70	73.2
71	71	74.4
72	72	75.6
73	73	76.8
74	74	78
75	75	79.2
76	76	80.4
77	77	81.6
78	78	82.8
79	79	84
80	80	85.2
81	81	86.4
82	82	87.6
83	83	88.8
84	84	90
85	85	91.2
86	86	92.4
87	87	93.6
88	88	94.8
89	89	96
90	90	97.2
91	91	98.4
92	92	99.6
93	93	100.8
94	94	102
95	95	103.2
96	96	104.4
97	97	105.6
98	98	106.8
99	99	108
100	100	109.2

Table A13: Cholesky decomposition matrix for model in Table A1a (non-fatal CHD)

	Age	SIMD sc.	Diabetes	Fam. his.	CPD	SBP	TC	HDL	Constant	Gamma
Age	1.26E-05									
SIMD sc.	-5.63E-08	1.67E-06								
Diabetes	-1.9E-05	-9.68E-06	0.03375							
Fam. his.	1.32E-05	-1.65E-06	0.000132	0.003352						
CPD	8.88E-07	-4.29E-07	2.49E-05	-7.14E-07	4.76E-06					
SBP	-1.36E-06	-8.92E-08	-9.51E-06	-2.38E-06	5.41E-08	1.97E-06				
TC	-1.82E-06	2.97E-06	-0.00025	-6.5E-05	-1.65E-06	-3.02E-06	0.000577			
HDL	-1.9E-05	4.02E-06	0.000214	8.21E-06	1.71E-05	-2.24E-06	-0.00022	0.008855		
Constant	-0.00045	-5.9E-05	0.002371	-0.00105	-0.0001	-0.00018	-0.00306	-0.0087	0.086123	
Gamma	1.54E-06	6.02E-07	4.04E-05	4.01E-06	5.24E-07	2.54E-07	2.35E-06	-2.1E-05	-0.00034	1.76E-05

Table A14: Cholesky decomposition matrix for model in Table A1a (non-fatal CBVD)

	Age	SIMD sc.	Diabetes	Fam. his.	CPD	SBP	TC	HDL	Constant	Gamma
Age	3.79E-05									
SIMD sc.	-1.01E-06	4.46E-06								
Diabetes	-5.6E-05	-3.3E-05	0.065828							
Fam. his.	3.75E-05	-4.72E-06	0.000427	0.011873						
CPD	3.28E-06	-1.23E-06	7.57E-05	-7.44E-06	1.32E-05					
SBP	-3.95E-06	-3.16E-07	-1E-05	-1.2E-05	5.24E-08	4.98E-06				
TC	-7.37E-06	9.44E-06	-0.00024	-0.00017	-1.20E-07	-7.77E-06	0.001865			
HDL	-3E-05	3.05E-06	0.002974	-0.00029	2.43E-05	-1.8E-05	-0.00071	0.018683		
Constant	-0.00143	-0.00011	-0.002	-0.00161	-0.00033	-0.00041	-0.00964	-0.01667	0.241253	
Gamma	5.86E-06	1.87E-06	0.000121	6.77E-06	1.76E-06	7.97E-07	3.09E-06	-3.7E-05	-0.00118	5.37E-05

Table A15: Cholesky decomposition matrix for model in Table A1a (fatal CVD)

	Age	SIMD sc.	Diabetes	Fam. his.	CPD	SBP	TC	HDL	Constant	Gamma
Age	2.84E-05									
SIMD sc.	-1.41E-06	3.04E-06								
Diabetes	-2.9E-05	-2.2E-05	0.057734							
Fam. his.	2.89E-05	-4.29E-06	0.000287	0.007269						
CPD	2.89E-06	-8.25E-07	3.37E-05	-7.79E-06	8.31E-06					
SBP	-2.68E-06	-2.19E-07	-3.69E-06	-1E-05	-1.17E-07	2.82E-06				
TC	3.20E-06	6.64E-06	-0.00037	-7.8E-05	4.02E-06	-3.16E-06	0.001252			
HDL	-3E-05	-1.52E-07	-0.00018	-0.00031	7.41E-06	-2.5E-05	-0.0006	0.013304		
Constant	-0.00119	-3.2E-05	0.001989	-0.00097	-0.00026	-0.0002	-0.00717	-0.00859	0.165284	
Gamma	4.71E-06	1.12E-06	0.0001	1.81E-06	1.27E-06	4.50E-07	4.01E-06	-4E-05	-0.00078	3.56E-05

Table A16: Cholesky decomposition matrix for model in Table A1a (fatal non-CVD)

	Age	SIMD sc.	Diabetes	Fam. his.	CPD	SBP	TC	HDL	Constant	Gamma
Age	1.83E-05									
SIMD sc.	-7.87E-07	2.10E-06								
Diabetes	-1.7E-05	-1.3E-05	0.06594							
Fam. his.	1.73E-05	-2.54E-06	0.000451	0.005661						
CPD	1.72E-06	-5.79E-07	3.27E-05	-3.54E-06	5.78E-06					
SBP	-1.63E-06	-1.57E-07	-4.59E-06	-5.03E-06	7.55E-08	2.71E-06				
TC	-2.47E-06	4.63E-06	-0.00014	-8E-05	1.39E-06	-4.19E-06	0.000922			
HDL	-1.6E-05	1.42E-06	0.001089	-1.2E-05	8.01E-06	-9.23E-06	-0.00024	0.007462		
Constant	-0.00076	-3.8E-05	-0.00081	-0.00098	-0.00018	-0.00024	-0.00479	-0.00697	0.123588	
Gamma	3.27E-06	8.97E-07	3.96E-05	1.28E-06	8.05E-07	3.28E-07	2.69E-07	-1.2E-05	-0.00056	2.51E-05

Table A17: Cholesky decomposition matrix for model in Table A1b (non-fatal CHD)

	Age	SIMD sc.	Diabetes	Fam. his.	CPD	SBP	TC	HDL	Constant	Gamma
Age	2.18E-05									
SIMD sc.	-7.96E-07	2.26E-06								
Diabetes	-2.9E-05	-9.53E-06	0.037997							
Fam. his.	0.000018	-2.08E-06	8.26E-05	0.004177						
CPD	2.66E-06	-8.93E-07	2.72E-05	-1.58E-06	9.93E-06					
SBP	-2.46E-06	-1.20E-07	-2.51E-06	-4.40E-06	2.39E-07	2.50E-06				
TC	-3.7E-05	5.10E-06	-0.00027	-0.00013	-1.2E-05	-3.42E-06	0.000695			
HDL	-3.7E-05	3E-05	0.00214	-4E-05	3.5E-05	3.95E-06	-0.00022	0.009329		
Constant	-0.00054	-9.9E-05	-0.00115	-0.00131	-0.00021	-0.00019	-0.00201	-0.01228	0.100345	
Gamma	2.87E-06	8.61E-07	3.61E-05	8.54E-06	7.60E-07	1.70E-07	-2.70E-06	-3.3E-05	-0.00048	2.55E-05

Table A18: Cholesky decomposition matrix for model in Table A1b (non-fatal CBVD)

	Age	SIMD sc.	Diabetes	Fam. his.	CPD	SBP	TC	HDL	Constant	Gamma
Age	5.7E-05									
SIMD sc.	-3.01E-06	5.43E-06								
Diabetes	-8.7E-05	-3E-05	0.066499							
Fam. his.	5.36E-05	-3.65E-06	0.000852	0.011053						
CPD	7.60E-06	-2.08E-06	5.76E-05	2.80E-06	2.46E-05					
SBP	-5.89E-06	-3.39E-07	-1.1E-05	-4.99E-06	4.55E-07	5.78E-06				
TC	-0.00012	1.56E-05	-0.00029	-0.00044	-2.7E-05	-1.4E-05	0.002499			
HDL	-9.3E-05	6.54E-05	0.003344	0.00022	8.84E-05	3.79E-06	-0.00045	0.021592		
Constant	-0.00143	-0.0002	-0.00087	-0.00427	-0.00059	-0.00041	-0.00758	-0.02873	0.264345	
Gamma	9.61E-06	2.25E-06	8.87E-05	2.39E-05	2.09E-06	4.86E-07	-1.4E-05	-7.9E-05	-0.0014	6.83E-05

Table A19: Cholesky decomposition matrix for model in Table A1b (fatal CVD)

	Age	SIMD sc.	Diabetes	Fam. his.	CPD	SBP	TC	HDL	Constant	Gamma
Age	5.22E-05									
SIMD sc.	-3.12E-06	4.91E-06								
Diabetes	-6.5E-05	-3E-05	0.056007							
Fam. his.	4.58E-05	-1.64E-06	0.000961	0.009241						
CPD	6.60E-06	-1.85E-06	3.83E-05	9.60E-06	1.48E-05					
SBP	-4.81E-06	-1.95E-07	-2.57E-06	-4.48E-06	4.62E-07	4.30E-06				
TC	-7.1E-05	1.13E-05	-0.00014	-0.00025	-2E-05	-5.45E-06	0.001614			
HDL	-4.1E-05	4.73E-05	0.002741	0.000329	5.4E-05	3.09E-06	-0.00069	0.015663		
Constant	-0.00173	-0.00012	-0.00286	-0.0045	-0.00049	-0.00032	-0.00528	-0.0204	0.241351	
Gamma	8.99E-06	1.83E-06	8.77E-05	2.29E-05	1.31E-06	4.95E-07	-5.91E-06	-5.4E-05	-0.00128	5.58E-05

Table A20: Cholesky decomposition matrix for model in Table A1b (fatal non-CVD)

	Age	SIMD sc.	Diabetes	Fam. his.	CPD	SBP	TC	HDL	Constant	Gamma
Age	2.61E-05									
SIMD sc.	-1.22E-06	2.61E-06								
Diabetes	-5.4E-05	-1.3E-05	0.103373							
Fam. his.	1.88E-05	-1.43E-06	0.000119	0.005609						
CPD	3.22E-06	-1.10E-06	1.32E-05	2.35E-06	8.98E-06					
SBP	-2.87E-06	-1.45E-07	-8.82E-06	-3.44E-06	2.02E-07	3.01E-06				
TC	-4.9E-05	5.38E-06	0.000137	-0.00016	-1.3E-05	-4.92E-06	0.00103			
HDL	-2.1E-05	2.4E-05	0.000733	0.000174	3.07E-05	7.74E-07	-0.00041	0.008691		
Constant	-0.0007	-7.7E-05	0.000422	-0.00161	-0.00022	-0.00022	-0.00277	-0.01125	0.118397	
Gamma	3.99E-06	1.10E-06	4.08E-05	8.37E-06	5.54E-07	1.90E-07	-4.73E-06	-2.5E-05	-0.00062	3.03E-05

Table A21: Cholesky decomposition matrix for model in Table A2a

	Age at event	SIMD score	Family history	Constant	Gamma
Age at event	3.95E-05				
SIMD score	1.29E-06	4.53E-06			
Family history	5.81E-05	-1.1E-05	0.01014		
Constant	-0.00275	-0.00023	-0.00676	0.202488	
Gamma	2.62E-05	2.19E-06	1.38E-05	-0.00246	0.000116

Table A22: Cholesky decomposition matrix for model in Table A2b

	Age at event	SIMD score	Family history	Constant	Gamma
Age at event	9.59E-05				
SIMD score	2.70E-06	9.60E-06			
Family history	0.000123	6.25E-07	0.027277		
Constant	-0.00685	-0.00052	-0.01452	0.513441	
Gamma	7.25E-05	4.20E-06	-7.7E-05	-0.00675	0.000379

Table A23: Cholesky decomposition matrix for model in Table A2c

	Age at event	SIMD score	Family history	Constant	Gamma
Age at event	6.08E-05				
SIMD score	8.05E-07	6.06E-06			
Family history	5.03E-05	-4.91E-06	0.014259		
Constant	-0.00437	-0.00026	-0.00863	0.3313	
Gamma	4.5E-05	4.42E-07	-6.3E-05	-0.00411	0.000197

Table A24: Cholesky decomposition matrix for model in Table A2d

	Age at event	SIMD score	Family history	Constant	Gamma
Age at event	0.000106				
SIMD score	-2.60E-06	1.41E-05			
Family history	0.000242	-2.6E-05	0.028796		
Constant	-0.00775	-0.00031	-0.02781	0.604536	
Gamma	9.8E-05	7.31E-07	6.96E-05	-0.00917	0.000503

Table A25: Cholesky decomposition matrix for model in Table A5a (CHD)

	ti1	ti2	Age	SIMD sc.	Fam. his.	Constant
ti1	0.000116					
ti2	-0.00014	0.000182				
Age	6.22E-06	-3.90E-06	8.74E-06			
SIMD sc.	1.35E-06	-1.27E-06	7.33E-07	9.95E-07		
Fam. his.	-6.39E-06	9.07E-06	-2.52E-07	-4.01E-06	0.001949	
Constant	-0.00085	0.000739	-0.00059	-7.7E-05	-0.00052	0.043191

Table A26: Cholesky decomposition matrix for model in Table A5a (Stroke)

	ti1	ti2	Age	SIMD sc.	Fam. his.	Constant
ti1	0.00055					
ti2	-0.00071	0.00104				
Age	1.71E-05	-1.2E-05	1.69E-05			
SIMD sc.	4.22E-06	-5.92E-06	1.17E-07	2.90E-06		
Fam. his.	-0.00018	0.000272	-3.8E-05	-6.14E-06	0.006407	
Constant	-0.00273	0.002691	-0.00109	-9.8E-05	0.000618	0.080138

Table A27: Cholesky decomposition matrix for model in Table A5a (Intermittent claudication)

	ti1	ti2	Age	SIMD sc.	Fam. his.	Constant
ti1	0.000791					
ti2	-0.001	0.001377				
Age	1.76E-05	-1.5E-05	3.32E-05			
SIMD sc.	-1E-05	1.48E-05	8.78E-07	5.65E-06		
Fam. his.	0.000179	-5.8E-05	7.24E-06	4.25E-05	0.011861	
Constant	-0.00315	0.003197	-0.00213	-0.00022	-0.00647	0.152848

Table A28: Cholesky decomposition matrix for model in Table A5a (Other heart condition)

	ti1	ti2	Age	SIMD sc.	Fam. his.	Constant
ti1	0.000255					
ti2	-0.00032	0.000457				
Age	1.94E-06	6.99E-06	1.42E-05			
SIMD sc.	-9.29E-07	5.15E-07	2.97E-07	1.73E-06		
Fam. his.	-2.8E-05	6.41E-05	2.23E-06	-5.36E-06	0.003964	
Constant	-0.00093	0.000466	-0.00091	-6E-05	-0.00128	0.063427

Table A29: Cholesky decomposition matrix for model in Table A5a (Heart failure)

	ti1	ti2	Age	SIMD sc.	Fam. his.	Constant
ti1	0.000474					
ti2	-0.00065	0.001032				
Age	3.61E-06	9.15E-06	2.24E-05			
SIMD sc.	-4.88E-06	5.68E-06	1.18E-06	3.65E-06		
Fam. his.	-0.00037	0.000592	6.46E-05	-1.9E-05	0.007119	
Constant	-0.00115	0.000503	-0.00148	-0.00016	-0.00552	0.104626

Table A30: Cholesky decomposition matrix for model in Table A5b (CHD)

	ti1	ti2	Age	SIMD sc.	Fam. his.	Constant
ti1	0.001704					
ti2	-0.00223	0.003231				
Age	1.06E-05	2.87E-05	4.43E-05			
SIMD sc.	-1.5E-05	3.77E-05	8.41E-06	9.53E-06		
Fam. his.	5.77E-05	-0.00048	-0.00015	-2.8E-05	0.013653	
Constant	-0.00491	0.00292	-0.00298	-0.00075	0.005427	0.222996

Table A31: Cholesky decomposition matrix for model in Table A5b (Stroke)

	ti1	ti2	Age	SIMD sc.	Fam. his.	Constant
ti1	0.000699					
ti2	-0.00095	0.001526				
Age	6.88E-06	2.44E-05	3.22E-05			
SIMD sc.	-1.94E-06	3.26E-06	2.00E-08	2.81E-06		
Fam. his.	0.000114	-0.00014	5.2E-05	-1.5E-05	0.007743	
Constant	-0.00247	0.000772	-0.00219	-7.9E-05	-0.0054	0.15928

Table A32: Cholesky decomposition matrix for model in Table A5b (Intermittent claudication)

	ti1	ti2	Age	SIMD sc.	Fam. his.	Constant
ti1	0.002869					
ti2	-0.00465	0.010147				
Age	-7.46E-07	0.000169	8.09E-05			
SIMD sc.	-7.5E-05	0.000103	9.39E-06	1.11E-05		
Fam. his.	-0.0059	0.012305	0.000554	0.000302	0.034519	
Constant	-0.00331	-0.0081	-0.00573	-0.00086	-0.04223	0.433269

Table A33: Cholesky decomposition matrix for model in Table A5b (Other heart condition)

	ti1	ti2	Age	SIMD sc.	Fam. his.	Constant
ti1	0.001072					
ti2	-0.00147	0.002248				
Age	-3E-05	5.94E-05	4.23E-05			
SIMD sc.	-4.57E-06	8.30E-06	1.61E-06	4.90E-06		
Fam. his.	6.3E-05	0.000233	0.000118	-1.9E-05	0.01287	
Constant	-0.00099	-0.0003	-0.00274	-0.00025	-0.01138	0.195118

Table A34: Cholesky decomposition matrix for model in Table A5b (Heart failure)

	ti1	ti2	Age	SIMD sc.	Fam. his.	Constant
ti1	0.005537					
ti2	-0.00661	0.008474				
Age	-1.4E-05	0.000129	0.000245			
SIMD sc.	-2E-05	1.41E-05	1.18E-05	3.26E-05		
Fam. his.	0.007062	-0.00838	0.00166	-0.00011	0.045189	
Constant	-0.01502	0.009929	-0.01775	-0.00146	-0.1455	1.360667

Table A35: Cholesky decomposition matrix for model in Table A5c (CHD)

	ti1	ti2	Age	SIMD sc.	Fam. his.	Constant
ti1	0.000234					
ti2	-0.00034	0.000556				
Age	7.30E-06	-1.41E-06	9.43E-06			
SIMD sc.	2.23E-07	1.53E-07	2.47E-07	1.24E-06		
Fam. his.	3.33E-05	-8.2E-05	2.36E-06	2.37E-06	0.002697	
Constant	-0.00133	0.001218	-0.00065	-5.9E-05	-0.0015	0.05111

Table A36: Cholesky decomposition matrix for model in Table A5c (Stroke)

	ti1	ti2	Age	SIMD sc.	Fam. his.	Constant
ti1	0.001357					
ti2	-0.00229	0.004284				
Age	1.93E-06	4.35E-05	4.09E-05			
SIMD sc.	-6.81E-06	7.28E-06	1.07E-06	3.73E-06		
Fam. his.	-0.00122	0.00209	-6.4E-05	1.87E-05	0.0112	
Constant	-0.00273	0.00131	-0.00266	-0.0002	0.000198	0.190148

Table A37: Cholesky decomposition matrix for model in Table A5c (Stroke)

	ti1	ti2	Age	SIMD sc.	Fam. his.	Constant
ti1	0.001357					
ti2	-0.00229	0.004284				
Age	1.93E-06	4.35E-05	4.09E-05			
SIMD sc.	-6.81E-06	7.28E-06	1.07E-06	3.73E-06		
Fam. his.	-0.00122	0.00209	-6.4E-05	1.87E-05	0.0112	
Constant	-0.00273	0.00131	-0.00266	-0.0002	0.000198	0.190148

Table A38: Cholesky decomposition matrix for model in Table A5c (Intermittent claudication)

	ti1	ti2	Age	SIMD sc.	Fam. his.	Constant
ti1	0.001435					
ti2	-0.00218	0.003827				
Age	9.26E-05	-9.4E-05	7.39E-05			
SIMD sc.	3.25E-05	-5.5E-05	1.08E-05	1.07E-05		
Fam. his.	-0.00129	0.002581	-6.1E-05	-0.00018	0.016355	
Constant	-0.01065	0.012432	-0.00549	-0.00104	0.005723	0.429304

Table A39: Cholesky decomposition matrix for model in Table A5c (Other heart condition)

	ti1	ti2	Age	SIMD sc.	Fam. his.	Constant
ti1	0.000458					
ti2	-0.00073	0.001295				
Age	-1.1E-05	3.1E-05	2.07E-05			
SIMD sc.	7.57E-06	-1.3E-05	-1.92E-06	2.57E-06		
Fam. his.	-0.00012	0.000291	8.01E-05	-2.3E-05	0.004562	
Constant	-0.00086	0.000269	-0.00131	3.36E-05	-0.00646	0.092703

Table A40: Cholesky decomposition matrix for model in Table A5c (Heart failure)

	ti1	ti2	Age	SIMD sc.	Fam. his.	Constant
ti1	0.000852					
ti2	-0.0013	0.002279				
Age	2.99E-05	-2.79E-06	3.4E-05			
SIMD sc.	-5.12E-06	1.1E-05	-6.32E-07	5.37E-06		
Fam. his.	0.000209	-8.1E-05	0.0002	-4.3E-05	0.011249	
Constant	-0.00414	0.002906	-0.00239	-0.00012	-0.01719	0.183767

Table A41: Cholesky decomposition matrix for model in Table A5d (CHD)

	ti1	ti2	Age	SIMD sc.	Fam. his.	Constant
ti1	0.002913					
ti2	-0.0036	0.004895				
Age	5.66E-05	-3.5E-05	4.96E-05			
SIMD sc.	2.5E-05	-3.4E-05	4.63E-06	1.24E-05		
Fam. his.	-0.00075	0.000935	0.000256	3.36E-05	0.02192	
Constant	-0.01391	0.014156	-0.00366	-0.00082	-0.02412	0.327691

Table A42: Cholesky decomposition matrix for model in Table A5d (Stroke)

	ti1	ti2	Age	SIMD sc.	Fam. his.	Constant
ti1	0.000747					
ti2	-0.00099	0.001517				
Age	6.09E-06	1.84E-05	1.9E-05			
SIMD sc.	-5.94E-06	1.05E-05	2.83E-07	2.86E-06		
Fam. his.	-1.1E-05	-5.6E-05	6.19E-05	-5.67E-06	0.005877	
Constant	-0.0024	0.000977	-0.00136	-0.00011	-0.00624	0.109062

Table A43: Cholesky decomposition matrix for model in Table A5d (Intermittent claudication)

	ti1	ti2	Age	SIMD sc.	Fam. his.	Constant
ti1	0.005439					
ti2	-0.00724	0.011061				
Age	0.000127	-4.25E-06	0.000106			
SIMD sc.	0.00021	-0.00011	3.52E-05	4.52E-05		
Fam. his.	-0.00985	0.017791	0.000734	0.000311	0.063795	
Constant	-0.02696	0.014837	-0.00872	-0.00495	-0.05998	0.871108

Table A44: Cholesky decomposition matrix for model in Table A5d (Other heart condition)

	ti1	ti2	Age	SIMD sc.	Fam. his.	Constant
ti1	0.001881					
ti2	-0.00226	0.002897				
Age	2.12E-06	3.08E-05	3.17E-05			
SIMD sc.	-1.8E-05	2.22E-05	7.60E-07	5.82E-06		
Fam. his.	-0.00051	0.000802	1.61E-06	8.43E-06	0.014557	
Constant	-0.0045	0.002861	-0.00213	-0.00021	-0.00687	0.168674

Table A45: Cholesky decomposition matrix for model in Table A5d (Heart failure)

	ti1	ti2	Age	SIMD sc.	Fam. his.	Constant
ti1	0.004548					
ti2	-0.00526	0.006544				
Age	0.000148	-0.00012	6.26E-05			
SIMD sc.	0.000108	-1E-04	8.03E-06	1.95E-05		
Fam. his.	-0.00054	-6.5E-05	0.000191	-0.00013	0.040833	
Constant	-0.02545	0.024304	-0.00488	-0.0015	-0.02375	0.466341

Table A46: Cholesky decomposition matrix for model in Table A6a (non-fatal CHD)

	ti1	ti2	Age	SIMD sc.	Fam. his.	Constant
ti1	110.6293					
ti2	-223.332	520.3348				
Age	-1.84E+00	9.87E+00	10.46827			
SIMD sc.	5.00E-01	-1.26E+00	-2.78E-02	1.26E+00		
Fam. his.	69.02028	-158.226	-18.3664	7.62E-01	2217.569	
Constant	-299.578	260.9194	-512.02	-29.9892	73.61406	27551.52

Table A47: Cholesky decomposition matrix for model in Table A6a (non-fatal CBVD)

	ti1	ti2	Age	SIMD sc.	Fam. his.	Constant
ti1	491.8385					
ti2	-917.366	1862.982				
Age	-20.948	52.19749	34.82615			
SIMD sc.	-1.58E+00	6.72E+00	2.62E+00	3.540867		
Fam. his.	-4.58151	-139.728	35.95839	-22.1553	7226.995	
Constant	-388.898	-93.4803	-1881.86	-219.977	-3933.45	110094.3

Table A48: Cholesky decomposition matrix for model in Table A6a (fatal CVD)

	ti1	ti2	Age	SIMD sc.	Fam. his.	Constant
ti1	287.3269					
ti2	-454.53	871.4238				
Age	2.54E+01	-4.33E+01	32.50656			
SIMD sc.	-1.55E+00	4.19E+00	-7.13E-01	4.10E+00		
Fam. his.	-282.262	699.2308	-11.8609	2.43E+00	9225.866	
Constant	-2225.1	3458.229	-1784.69	-65.4644	-1368.86	105276.2

Table A49: Cholesky decomposition matrix for model in Table A6a (fatal non-CVD)

	ti1	ti2	Age	SIMD sc.	Fam. his.	Constant
ti1	564.796					
ti2	-877.975	1671.553				
Age	1.22E+01	-3.08E+01	64.52869			
SIMD sc.	-2.37E-01	-1.95E+00	-1.13E-01	7.18E+00		
Fam. his.	-260.844	-159.267	258.182	-3.68E+01	25937.41	
Constant	-2428.75	4335.331	-3399.22	-192.728	-14825.5	192596.7

Table A50: Cholesky decomposition matrix for model in Table A6b (non-fatal CHD)

	ti1	ti2	Age	SIMD sc.	Fam. his.	Constant
ti1	1974.068					
ti2	-5329.28	14590.2				
Age	5.34E+02	-1.46E+03	170.6693			
SIMD sc.	-1.73E+02	4.80E+02	-4.88E+01	1.82E+01		
Fam. his.	-12380.7	34248.04	-3423.89	1148.822	85633.54	
Constant	-23205.6	62879.94	-7557.32	2.03E+03	143852	341257.6

Table A51: Cholesky decomposition matrix for model in Table A6b (non-fatal CBVD)

	ti1	ti2	Age	SIMD sc.	Fam. his.	Constant
ti1	364.5978					
ti2	-561.626	1067.69				
Age	8.484686	-4.48079	32.95695			
SIMD sc.	-17.6807	1.65E+01	3.90E+00	9.261196		
Fam. his.	92.03296	257.3483	-1.43E+01	-1.17E+02	10339.79	
Constant	-1482.85	1622.071	-1865.68	-286.699	-1921.16	113819.8

Table A52: Cholesky decomposition matrix for model in Table A6b (fatal CVD)

	ti1	ti2	Age	SIMD sc.	Fam. his.	Constant
ti1	466.4651					
ti2	-731.87	1370.365				
Age	3.61E+01	-37.9897	61.01627			
SIMD sc.	-6.03E+00	1.18E+01	-1.20E+00	5.79E+00		
Fam. his.	-232.879	704.4573	2.46E+02	1.48E+01	13827.75	
Constant	-3418.26	3871.214	-3528.06	-108.431	-18499.7	218775.7

Table A53: Cholesky decomposition matrix for model in Table A6b (fatal non-CVD)

	ti1	ti2	Age	SIMD sc.	Fam. his.	Constant
ti1	585.1934					
ti2	-860.987	1534.322				
Age	-1.27E+01	-6.08E-01	98.38825			
SIMD sc.	-2.75E+00	7.90E+00	-1.83E+00	1.03E+01		
Fam. his.	194.8229	-458.174	211.0032	-45.4992	20036.14	
Constant	-1298.57	2611.277	-5204.9	-173.528	-16101.9	293698.5

Table A54: Cholesky decomposition matrix for model in Table A6c

	ti1	ti2	Age	SIMD sc.	Fam. his.	Constant
ti1	1928.527					
ti2	-2182.32	2623.261				
Age	-6.64E+01	9.01E+01	81.79826			
SIMD sc.	-8.98E+00	8.84E+00	-3.28E-01	1.13E+01		
Fam. his.	-1664.72	1803.166	206.9212	-44.8729	26691.6	
Constant	-4767.18	4054.117	-4563.5	-212.089	-9528.91	305362.2

Table A55: Cholesky decomposition matrix for model in Table A6d

	ti1	ti2	Age	SIMD sc.	Fam. his.	Constant
ti1	7948.634					
ti2	-9946.35	14008.03				
Age	3.33E+01	145.9542	256.7264			
SIMD sc.	1.51E+01	-4.41E+01	-1.55E+00	3.30E+01		
Fam. his.	-1305.24	3012.042	304.1178	-603.492	98476.88	
Constant	-33236	25913.37	-16488.6	-701.861	-24693.6	1230386

Table A56: Cholesky decomposition matrix for model in Table A6e

	ti1	ti2	Age	SIMD sc.	Fam. his.	Constant
ti1	2803.303					
ti2	-3796.26	5472.314				
Age	57.77047	-23.4372	128.3375			
SIMD sc.	2.89E+01	-3.10E+01	5.64E+00	1.52E+01		
Fam. his.	961.1155	-1232.25	781.685	-58.8688	35371.38	
Constant	-16976.6	18323.64	-8480.98	-900.972	-64442.5	648552.4

Table A57: Cholesky decomposition matrix for model in Table A6f

	ti1	ti2	Age	SIMD sc.	Fam. his.	Constant
ti1	10606.79					
ti2	-12042.9	14774.05				
Age	2.41E+02	-60.7626	235.9434			
SIMD sc.	1.87E+01	-1.30E+01	7.65E+00	3.95E+01		
Fam. his.	2558.769	-3268.72	848.302	-1.60E+02	82288.52	
Constant	-58715	50174.18	-16693.2	-1820.52	-96858.7	1428460

Figure A1: Observed proportions of HF hospitalisations over time since first event for men (circles = proportions following non-fatal CHD event, squares = proportions following non-fatal CBVD event)

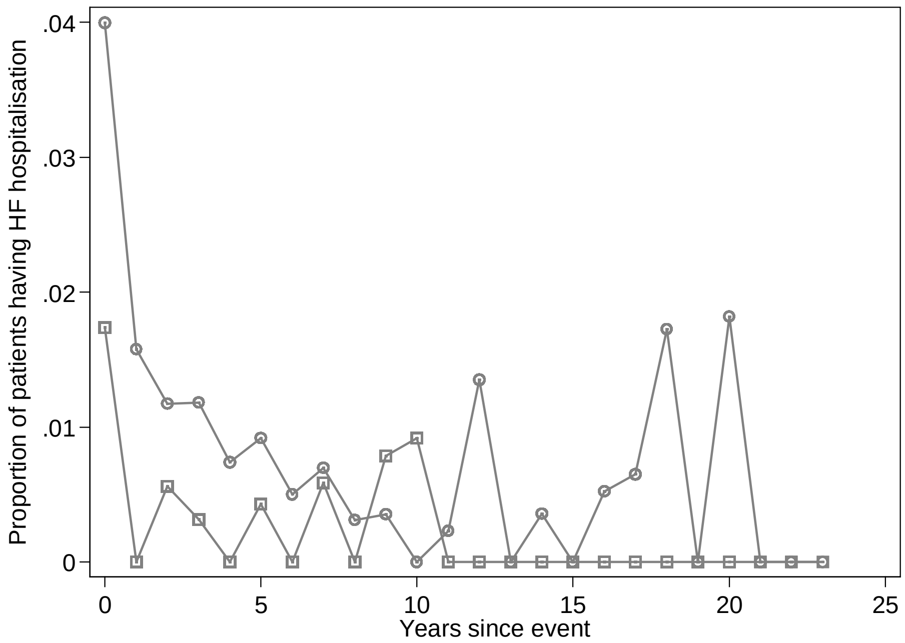

Figure A2: Observed mean costs over time since screening for men (circles = costs before non-fatal CHD event, squares = costs before non-fatal CBVD event, triangles = costs before fatal CVD event, crosses = costs before fatal non-CVD event)

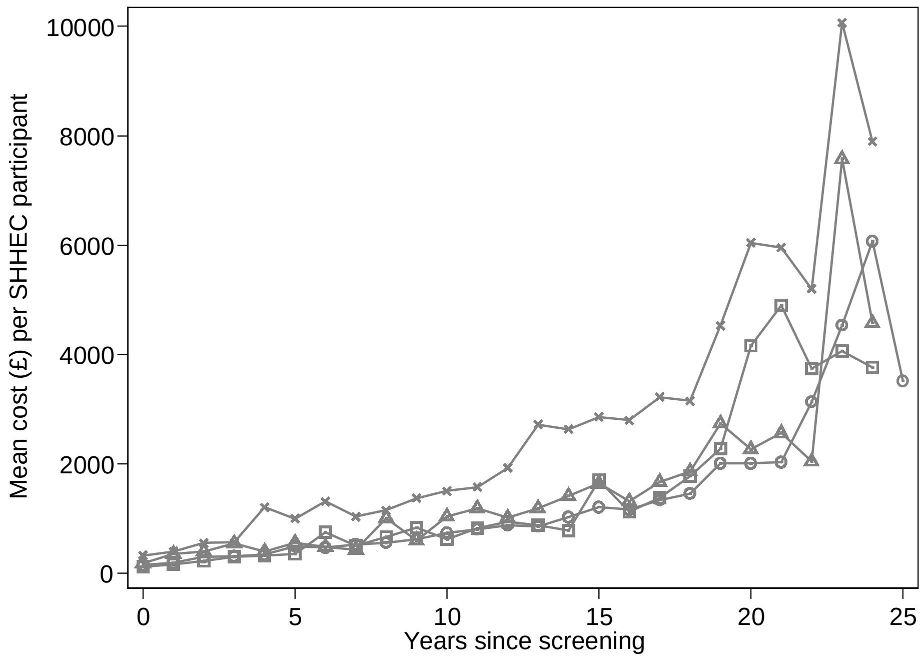

Figure A3: Observed mean costs over time since first event for men (circles = costs after non-fatal CHD event, squares = costs after non-fatal CBVD event)

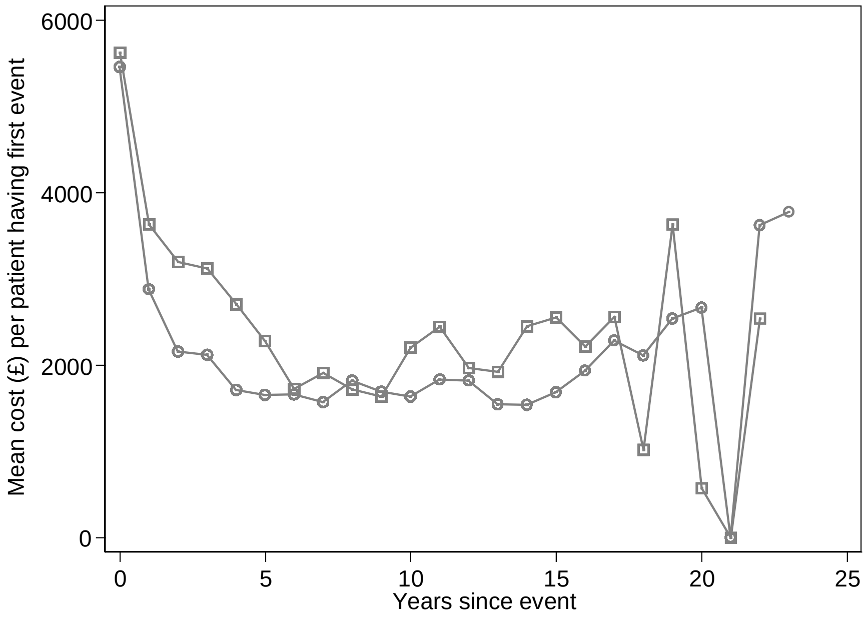

REFERENCES

1. **Lewsey JD**, Lawson KD, Ford I, *et al.* A Cardiovascular Disease Policy Model for Scotland: Part 1 – predicting life expectancy accounting for socio-economic deprivation.

2. **Lawson KD**, Lewsey JL, Ford I, *et al.* A Cardiovascular Disease Policy Model for Scotland: Part 2 – preparing for economic evaluation.

3. **Putter H**, Fiocco M and Geskus RB. Tutorial in biostatistics: Competing risks and multi-state models. *Statistics in Medicine* 2007;**26**:2389-2430.

4. **Clarke PM**, Hayes AJ, Glasziou PG, *et al.* Using the EQ-5D index score as a predictor of outcomes in patients with type 2 diabetes. *Medical Care* 2009;**47**:61-8.

5. **Harrell FE, Jr**. Regression Modeling Strategies: With Applications to Linear Models, Logistic Regression, and Survival Analysis. New York: Springer, 2001, p23.
